# Cognitive Matching of Design Subjects in Product Form Evolutionary Design

**DOI:** 10.1155/2021/8456736

**Published:** 2021-09-30

**Authors:** Shutao Zhang, Shijie Wang, Aimin Zhou, Shifeng Liu, Jianning Su

**Affiliations:** School of Design Art, Lanzhou University of Technology, Lanzhou 730050, China

## Abstract

In actual product development, the cognitive differences between users and designers make it difficult for the designed products to be recognized by users. To reduce the cognitive differences between these two design subjects, this paper proposes a method of cognitive matching of the design subjects. First, we use the relevant methods of Kansei engineering to quantify the Kansei image cognition of the two design subjects and construct a cognitive matching model of the design subjects with information entropy and the technique for order preference by similarity to ideal solution (TOPSIS). Second, according to the Kansei image, the Kansei image prototype cluster is constructed, and the representative Kansei image prototype is obtained. Then, we combine an artificial neural network (ANN) with a cognitive matching model of the design subjects to construct a product Kansei image evaluation system; this is used to evaluate the evolved forms. Finally, a product Kansei image form evolution system is constructed based on the genetic algorithm (GA). To some extent, the system simulates the cognitive matching process between designers and users in product design, helps designers to more accurately understand the cognitive trends of the two design subjects, and provides a theoretical basis for the intelligent design of product forms through the cognitive balance of multiple design subjects. This paper takes a beverage bottle as an example to verify the feasibility of the model through a comparative study.

## 1. Introduction

Design is a creative process of expressing human needs in concrete product forms, and its main purpose is to endow things with new value [1]. As a medium of the expression of designers' ideas and multilevel symbolic meaning, the creative product form is the optimal solution for expressing users' subjective needs through the characteristics of shape, function, and material. With the development of science and technology, product development technology has gradually transformed from traditional design and development methods to intelligent design modes based on computer technology, and the focus of product design has evolved from functional requirements to user-centred emotional satisfaction.

In product design, designers, users, and products are the three most important aspects. Designers and users are the core subjects of design. Designers infuse their own cognition into the product form through communication with users to meet the emotional needs of users [2]. However, there are very large differences between designers and users in terms of knowledge, educational background, personality characteristics, and understanding of the product. Therefore, by extracting Kansei variables related to product design elements, we can transform users' emotional characteristics into product attributes and accurately grasp and understand the Kansei image cognition trends between users and designers, which can help designers construct product forms that meet users' needs [3]. For example, K. Qiu et al. used Theil entropy to evaluate the cognitive friction between users and designers and built a cognitive friction balance model to alleviate the cognitive friction between the two groups [4]. Based on double coding in psychology, Hu et al. decomposed the cognition of automobile modelling Kansei images to compare the differences between users and designers in semantic word selection and corresponding morphological features [5].

In the traditional design process, designers use sketches to express the product scheme, which is tedious and requires strong drawing ability. The emergence of intelligent design provides new theories and methods for product form design which greatly change the task that designers undertake in the design process and provide a new way to shorten the design cycle. For example, Hsiao and Tsai constructed an automatic design system using a fuzzy neural network and genetic algorithm (GA) and made a preliminary attempt at intelligent product design [6]. As the core of intelligent design, evolutionary algorithms have been widely used in product design and have become one of the main means of product form evolution and evaluation [7]. Common evolutionary algorithms usually take the existing optimal design scheme as the parent sample for the genetic operation and generate a large number of new schemes. This method rapidly produces a large number of design schemes and also produces new problems; that is, with increasing evolutionary generation, the Kansei images of the product forms of many offspring gradually deviate from the target Kansei image. In reality, the Kansei image cognition of human beings has corresponding product form characteristics. We concretely express the Kansei image information of consumers to produce the product form representing the target Kansei image and call it the Kansei image prototype [8]. Combining the Kansei image prototype and the design scheme that needs to evolve, and with the offspring inheriting the characteristics of the parents, we take the morphological elements of the Kansei image prototype to lead the evolution of the offspring product form to help ensure that the offspring product form has the characteristics of the target Kansei image and always evolves towards the direction of the target Kansei image. We call this process Kansei image traction.

In recent years, many scholars have conducted research on the Kansei image cognition of users and designers. For example, to study the Kansei image needs and personality preferences of users, Yan et al. used the semantic difference (SD) method and language variables to obtain the Kansei data of products to obtain the satisfaction in product evaluation [9]. Reference [10] used eye tracking technology to collect users' needs and obtained users' Kansei image cognition and objective evaluation data of products by precise methods to meet the needs of specific target users. Reference [11] constructed a cognitive model to explore the relationships between design subjects, the design process, and the cognitive process. Xue et al. constructed an integrated decision-making system of optimized product Kansei image design by using the methods of quantification-I theory and grey correlation analysis to simulate the Kansei image evaluation of products by users to optimize the product form [12]. To better retain the characteristics of target Kansei images in product families, Zhang et al. proposed a multicriteria decision system of products from Kansei images based on a logistic regression model [13]. Xu et al. studied the differences in product Kansei image cognition between users and designers by using the method of gene network comparison, which helped designers more accurately understand the implicit needs of users and improve the market recognition of products [14].

At present, the related research mainly explores the Kansei image cognition of a single design subject. Although there are studies on the Kansei image cognition of different design subjects, most of them focus on comparing the differences in the product Kansei image cognition among different design subjects, while there are relatively few studies on the quantitative description and matching in the Kansei image cognition of multiple design subjects. In addition, in product evolutionary design, there are few studies on Kansei image traction for optimizing product form. Therefore, integrating user cognition into product form and accurately grasping the cognitive relationship between design subjects to realize the cognitive matching of design subjects represent an important research direction.

Based on the above problems, this paper proposes a method of the cognitive matching of design subjects in product form evolutionary design and explores the matching relationship of Kansei image cognition between users and designers in the process of design scheme generation in a quantitative way. First, we apply the methods of Kansei engineering to explore the inherent trend of Kansei image cognition between users and designers and construct the cognitive matching model of design subjects. Second, we introduce product prototype theory and the Kansei image cognitive dynamic mechanism and establish a Kansei image prototype cluster. Next, we determine a representative Kansei image prototype and take it as external traction to lead product form evolution. Then, the product Kansei image evaluation system is constructed by combining an artificial neural network (ANN) with the cognitive matching model of design subjects to evaluate whether the product evolution form has reached a state of a strong match in the Kansei image cognition of the two design subjects. Finally, we build a product Kansei image evolution system based on a GA and verify the feasibility of the model by comparing the results. This study provides a quantitative method and new research ideas for Kansei image cognitive matching between users and designers and has guiding significance for the continuation of the product Kansei image style in the process of product form evolution.

The rest of this article is structured as follows.  Section 2 introduces the related theories such as cognitive differences, cognitive matching, and the technique for order preference by similarity to ideal solution (TOPSIS).  Section 3 introduces the basic process of the product form evolution method based on the cognitive matching of the design subjects.  Section 4 demonstrates the above process with a case.  Section 5 presents analysis and discussion of the results and shortcomings of this study. Finally, Section 6 is a summary of this paper and explains the significance of the model.

## 2. Related Theories

### 2.1. Cognitive Differences

**Figure 1:**
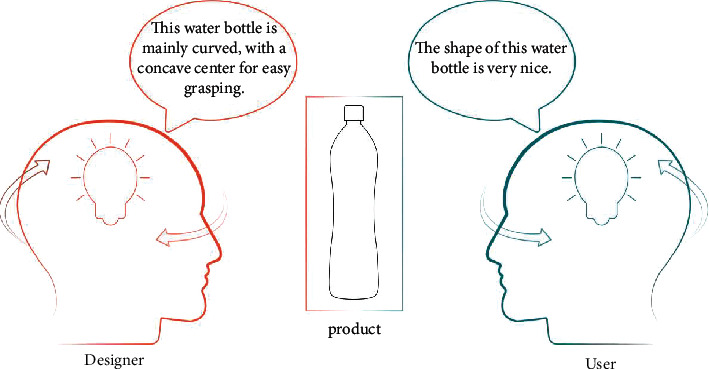
Map of cognitive differences.

Cognition is an information processing operation in which humans recognize objects in the environment through their senses and store the acquired object information in a structured form [15]. As the media of designers' cognitive expression, products communicate with users through their form, function, structure, and other aspects to convey designers' ideas. According to Maslow's hierarchy of needs theory, when people's basic needs are met, they pursue higher levels of needs [16]. With the continuous advancement of science and technology, emotional needs have gradually become the main factor affecting consumers' purchase of products. Lee and Chen used interactive qualitative analysis (IQA) to conduct psychological model analysis on different groups and explored the cognitive differences between different groups when using smartphones [17]. Reference [18] discussed the cognitive process of users on products and the differences between designers and users' mental models and proved that only products that conform to users' mental models can meet users' needs. Due to the influence of background factors, there are very large differences in the understanding of products of users and designers. That is, users experience imperfect cognition of their own class, personality, and knowledge and thus different understanding of the scenes needed by the products. Their cognition of products forms according to their own experience and knowledge. However, designers obtain a view of products from the aspects of demand mining, product positioning, product innovation and rationality, design expression, and other aspects. The two design subjects do not directly communicate, resulting in differences in the cognitive perception of the product, as shown in Figure 1.

The development of the market economy has promoted the gradual development of product design from meeting the needs of the public to customized experiences. The status of users in design has also been transformed from the recipients of products to participants in product design. Therefore, the cognitive differences between users and designers have become the focus of many scholars. For example, Yao and Huang abstracted multiple shape information into simple symbols based on the concept of abstract symbols and explored user cognition by establishing a mapping relationship between user cognition concepts and product shape information [19]. To prevent the failure of the user experience due to cognitive friction, He qualitatively analysed the causes and avoidance methods of cognitive friction in the process of product design and use [20]. Based on the clear perception characteristics of users and designers, [21] proposed a model of a team style-promoting design scheme in the face of perception conflict to explore the influence of the design team's perceived conflict behaviour style on the scheme process and product in interaction design.

The existing studies mostly focus on the qualitative exploration of the differences, factors, and influences between the two design subjects, while there are relatively few studies on the cognitive trends of users and designers in the process of product scheme generation. Therefore, this paper uses the quantitative method to study the trends of Kansei image cognition of the two design subjects in the process of product scheme generation.

### 2.2. Cognitive Matching

The user's cognition of a product mainly depends on the users' perception and the interaction process between them. With the continuous enrichment of the users' experience, their cognitive memories of products gradually strengthen. Under the stimulation of the cognitive system and external environment, users can perceive the connotation conveyed by a product when they contact the product. Even if they have never contacted the product form, users can generate cognitive structure according to their own cognitive memories, thus connecting the form elements with symbolic meaning [22]. In the actual production process, due to the differences in social responsibilities between users and designers, the two design subjects seldom directly communicate, meaning the product form is unable to reflect cognitive consistency between users and designers. To minimize cognitive differences, designers need to grasp the symbolic meaning perceived by users when they contact a product. According to the cognitive mode, designers construct a product form recognized by users and satisfy users' psychological expectations from the visual system to realize cognitive matching. Therefore, we can use computer technology for cognitive simulation to fully understand the cognitive processes of users and designers [23].

At present, cognitive matching is widely used in other fields. In the field of medicine, Zane et al. found cognitive differences between outpatients and clinicians in treatment and found that cognitive matching between them can affect the course of treatment and predict the treatment outcome [24]. To improve the inaccuracies in traditional clinical reasoning and the main cognitive biases in medicine, [25] established a cognitive balance model by using the diagnostic process of a fuzzy cognitive map. In the field of psychology, Roth et al. established a reclassification model based on cognitive consistency and explored whether the identity between individuals and groups determines the compatibility between different groups [26]. In the field of management, Hassan and Ralf studied human cognition and behaviour through the analysis of cognitive matching and predicted and discussed the use of accounting information [27]. In the field of design, to study the perception differences between users and designers in product modelling, [28] constructed a Kansei image matching model between users and designers. Su et al. developed a cobweb colliding evolutionary system of product form to study the cognitive balance between users and designers by simulating the design thinking of the cobweb structure and spider predation mode [29].

In summary, research on cognitive matching in different disciplines can solve the problems caused by cognitive bias to a certain extent. The related research in the field of design mainly focuses on exploring the cognitive matching of the two design subjects from the qualitative level, and there are relatively few quantitative studies on the cognitive trends of design subjects in the iterative process of a single scheme. Therefore, we introduce the cognitive matching mechanism into the product design process to quantitatively explore the cognitive variation in users and designers during product scheme iteration and propose a cognitive matching model.

### 2.3. TOPSIS Method

TOPSIS is a multicriteria decision analysis method that was developed by Ching-Lai Hwang and Yoon in 1981. The basic idea of this technique is to determine the optimal scheme (positive ideal solution) and the worst scheme (negative ideal solution) according to the original scheme and to compare the distances between all the original schemes and the optimal scheme and the worst scheme to judge the pros and cons of each scheme. The determined alternative scheme should be located at the shortest geometric distance from the optimal scheme and the longest geometric distance from the worst scheme [30]. As a multicriteria decision analysis method, TOPSIS has been successfully applied to industrial technology [31, 32], computer science [33–35], medical problems [36], applied statistics [37–40], environmental protection [41, 42], and other fields.

In the field of design, to address a design that is infeasible due to the uncertainty in customer preference in the cognition of the optimization goals, Zhou et al. used TOPSIS to rank factors such as production cost, time, product output, and pollution emissions [43]. Based on entropy theory and TOPSIS, Wang et al. proposed a comprehensive evaluation system with subjective and objective combinations to improve ergonomics evaluations and decision-making in product development [44]. To determine the best conceptual design in the process of concept evaluation, [45] introduced the customer evaluation and design specifications described by soft foundation theory and entropy theory into the TOPSIS framework to rank the pros and cons. On the basis of establishing the relationship between consumer demand and technical characteristics, [46] calculated the basic importance of technical characteristics and used the TOPSIS multiattribute decision model to modify the basic importance of technical characteristics to overcome the limitation of considering only consumer factors. Su et al. used the TOPSIS method to prioritize new samples coupled with different weight design elements to explore the coupling characteristics of elements in product Kansei image design [47].

TOPSIS is usually used to analyse the pros and cons of multiple indicators to select alternative indicators. In this study, the TOPSIS method is used to calculate the relative closeness between the Kansei image cognition and the target Kansei image of users and designers to obtain the coordination index of the two design subjects when they reach cognitive matching and to calculate the cognitive matching degree between the two design subjects.

## 3. Cognitive Matching of Design Subjects in Product Form Evolutionary Design

**Figure 2:**
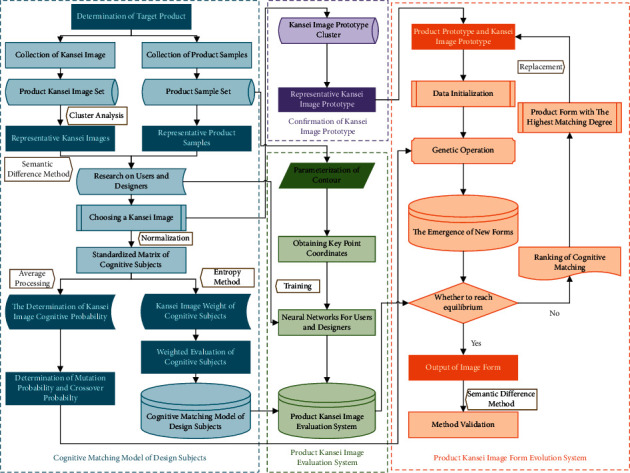
Research process.

The overall research process is divided into four parts, namely, the cognitive matching model of design subjects, determination of the Kansei image prototype, the product Kansei image evaluation system, and the product Kansei image form evolution system, as shown in Figure 2. First, we investigate the Kansei images of users and designers with representative samples and Kansei images and establish the standardized matrix of the two design subjects. Based on the entropy weight method and TOPSIS and other methods, we construct the cognitive matching model of the design subjects and determine the cognitive probability of the two design subjects by averaging the standardized matrix. Second, according to the selected Kansei image of user research, we obtain the product prototype representing the Kansei image. After screening, we construct the Kansei image prototype cluster and select the product prototype with the highest degree of recognition as the representative Kansei image prototype. Then, according to the corresponding relationship between the sample data and the Kansei image evaluation data, the Kansei image evaluation system of users and designers is constructed by using an ANN. Combined with the cognitive matching model of the design subjects, the product Kansei image evaluation system is constructed. Finally, we take the product prototype and representative Kansei image prototype as the parents for genetic operation. Through the judgement of the fitness function, we select the product form with the highest matching degree to replace the product prototype, repeat the genetic operation, and finally select the product form with high matching cognition. We conduct a cognitive investigation on the two design subjects to verify the feasibility of the model through comparative analysis.

### 3.1. Cognitive Matching Model of the Design Subjects

#### 3.1.1. Construction of the Evaluation Matrix of the Design Subjects

(1) *Determination of the Representative Product Samples.* We collect pictures of product samples from journals, websites, and other channels. To prevent visual interference from factors such as colours and patterns, we extract sample contours to establish the product sample set and determine representative samples by expert interviews.
(2) *Determination of the Representative Product Kansei Image.* The Kansei words of the target product are collected from journals, websites, and other channels. We can obtain the most representative Kansei words of the target product Kansei image from expert interviews and construct the Kansei image set; namely, *S* = [*s*_1_, *s*_2_,…, *s*_l_], where *S* is the Kansei image set and *s*_*l*_ is the Kansei image.
We use semantic similarity to investigate the Kansei word similarity of the experts, to establish the similarity evaluation matrix shown in formula (1), and to cluster the results to obtain the representative Kansei images.
  (1) $$\begin{array}{c} A=\left[\begin{array}{ccc} a_{11} & \cdots & a_{1l}\\ \vdots & \ddots & \vdots \\ a_{l1} & \cdots & a_{ll} \end{array}\right], \end{array}$$
where *A* is the similarity evaluation matrix of the Kansei words. *a*_*ll*_ is the similarity evaluation value.
(3) *Obtaining the Evaluation Matrix.* Combined with representative samples and Kansei images, the SD questionnaire is designed to evaluate Kansei image cognition. The commonly used SD questionnaire is divided into a 5-level scale and a 7-level scale. In this paper, the SD questionnaire of the 5-level scale is used to investigate the Kansei image cognition of the two design subjects, and the Kansei image matrix is constructed. The results are as follows:
  (2) $$\begin{array}{c} R^{\varphi }=\left[\begin{array}{c} \begin{array}{cc} r_{11}^{\varphi } & r_{12}^{\varphi } \end{array}\\ \begin{array}{cc} r_{21}^{\varphi } & r_{22}^{\varphi } \end{array}\\ \begin{array}{cc} \vdots & \vdots \end{array}\\ \begin{array}{cc} r_{i1}^{\varphi } & r_{i2}^{\varphi } \end{array} \end{array}\;\begin{array}{c} \begin{array}{cc} \cdots & r_{1j}^{\varphi } \end{array}\\ \begin{array}{cc} \cdots & r_{1j}^{\varphi } \end{array}\\ \begin{array}{cc} r_{xy}^{\varphi } & \vdots \end{array}\\ \begin{array}{cc} \cdots & r_{ij}^{\varphi } \end{array} \end{array}\;\right], \end{array}$$
where *R*^*φ*^ is the Kansei image recognition evaluation matrix. *φ* represents the design subject, and *φ* = 1 and 2 represent users and designers, respectively. *r*_*ij*_^*φ*^ is the evaluation value of the *φ*-th design subject on the *j*-th Kansei image of the *i*-th sample.

The *b*-th Kansei image is selected from the representative product Kansei images for further research, and the evaluation matrix for the *b*-th Kansei image is obtained as follows:

(3) $$\begin{array}{c} r_{b}^{\varphi }={\left[r_{1b}^{\varphi }r_{2b}^{\varphi }\cdots r_{ib}^{\varphi }\right]}^{T}, \end{array}$$

where *r*_*b*_^*φ*^ is the Kansei image cognitive evaluation matrix for the *b*-th Kansei image. *φ* represents the design subject, and *φ* = 1 and 2 represent users and designers, respectively. *r*_*ib*_^*φ*^ is the evaluation value of the *φ*-th design subject of the *i*-th sample of the *b*-th Kansei image.

To reduce the errors generated by the two design subjects in the evaluation process, we use formula (4) to normalize the cognitive evaluation matrix and to obtain the decision matrix *V*.

(4) $$\begin{array}{c} v_{ib}^{\varphi }=\frac{r_{ib}^{\varphi }-\min r_{ib}^{\varphi }}{\max r_{ib}^{\varphi }-\min r_{ib}^{\varphi }}, \end{array}$$

where *v*_*ib*_^*φ*^ is the normalized evaluation value of the *φ*-th design subject of the *i*-th sample of the *b*-th Kansei image.

#### 3.1.2. Determination of the Cognitive Probability

The decision matrix *V* is averaged to obtain the probability matrix *E*^*φ*^ of the Kansei image cognition between users and designers.

(5) $$\begin{array}{c} E^{\varphi }=\left[e_{b}^{\varphi }\right], \end{array}$$

where *e*_*b*_^*φ*^ is the cognitive probability of the *φ*-th design subject of the *b*-th Kansei image.

Because designers have received systematic design knowledge training, their cognition of products is mainly based on the feasibility of product solutions, and their cognition of products represents a small jump. However, due to the influence of complex factors such as educational background, users usually form their opinions based on their own needs, and there is a great leap in their cognition of a product. Therefore, the Kansei image cognitive probability of two design subjects is regarded by means of the crossover probability and the mutation probability to guide the subsequent evolution process of the product Kansei image form.

#### 3.1.3. Construction of the Cognitive Matching Model of the Design Subjects

Information entropy, proposed by Shannon in 1948, is a measure of the diversity or uniformity of microstates in thermodynamics. By calculating the entropy of the system, the probability of the system in this state can be judged; that is, the greater the entropy value is, the greater the probability is. The smaller the entropy value is, the smaller the probability is [48]. Some scholars have conducted in-depth research on information entropy, which provides a theoretical basis for the practical application of entropy theory. For example, Wan et al. [49–51] proposed fuzzy entropy, hesitant entropy, and cross-entropy to determine the weights in multiattribute group decision-making problems. Xu et al. [52] proposed the fuzzy entropy and hesitant entropy of probabilistic linguistic term sets (PLTSs) to calculate the total entropy of PLTSs and used the cross-entropy to determine the attribute weight of the decision system.

In recent years, information entropy has been introduced into the design field to explore the entropy values of various indicators of products [53]. This paper uses information entropy to analyse the Kansei image cognition of users and designers to determine the weight relationship between these two design subjects. The calculation formula of this entropy is as follows:

(6) $$\begin{array}{c} Q^{\varphi }=-k\overset{m}{\underset{i=1}{\sum }}P_{i}^{\varphi }\ln\;P_{i}^{\varphi }, \end{array}$$

where *Q*^*φ*^ is the entropy value of the *φ*-th design subject. *P*_*i*_^*φ*^ represents the proportion of the Kansei image cognitive probability of the *i*-th sample of the *φ*-th design subject in the Kansei image cognitive probability of the cognitive subject, and 0 < *P*_*i*_^*φ*^ < 1. *k* is a constant, 1/ln  *m*.

(1) Formula (7) is used to obtain the proportion of the Kansei image cognition probability of each sample in the Kansei image cognition probability of the design subject.
  (7) $$\begin{array}{c} P_{i}^{\varphi }=\frac{v_{ib}^{\varphi }}{\sum_{i=1}^{m}v_{ib}^{\varphi }}. \end{array}$$
(2) We substitute *P*_*i*_^*φ*^ into formula (6) to obtain the entropy value of the *φ*-th design subject, and the weight *W*^*φ*^ of the design subject in the evaluation process is
  (8) $$\begin{array}{c} W^{\varphi }=\frac{1-Q^{\varphi }}{\sum_{\varphi =1}^{n}\left(1-Q^{\varphi }\right)}, \end{array}$$
where *n* = 2.
(3) The Kansei image cognitive evaluation of users and designers is weighted to obtain the weighted cognitive evaluation value *Z*^*φ*^ of each design subject:
  (9) $$\begin{array}{c} Z^{\varphi }=W^{\varphi }{\bar{r}}_{b}^{\varphi }. \end{array}$$
(4) TOPSIS is introduced to calculate the relative closeness between the Kansei image cognition of the design subject and the target Kansei image; namely,
  (10) $$\begin{array}{c} t^{\varphi }=\frac{d^{\varphi -}}{d^{\varphi -}+d^{\varphi +}}, \end{array}$$
  (11) $$\begin{array}{c} d^{\varphi +}=\sqrt{\overset{m}{\underset{i=1}{\sum }}{\left(v_{ib}^{\varphi }-v_{b}^{\varphi +}\right)}^{2}}, \end{array}$$
  (12) $$\begin{array}{c} d^{\varphi -}=\sqrt{\overset{m}{\underset{i=1}{\sum }}{\left(v_{ib}^{\varphi }-v_{b}^{\varphi -}\right)}^{2}}, \end{array}$$
where *d*^*φ*+^ represents the Euclidean distance between the cognitive evaluation of the *φ*-th design subject and the cognitive evaluation of the optimal scheme. *d*^*φ*−^ represents the Euclidean distance between the cognitive evaluation of the *φ*-th design subject and the cognitive evaluation of the worst scheme. *v*_*b*_^*φ*+^ is expressed as the cognitive evaluation value of the optimal scheme of the *φ*-th design subject for the *b*-th Kansei image. *v*_*b*_^*φ*−^ is expressed as the cognitive evaluation value of the worst solution scheme of the *φ*-th design subject of the *b*-th Kansei image.
Thus, the coordination index *C* of the Kansei image cognition of the two design subjects is obtained:
  (13) $$\begin{array}{c} C=\frac{\prod_{\varphi =1}^{n}t^{\varphi }}{{\left(\sum_{\varphi =1}^{n}t^{\varphi }\right)}^{2}}. \end{array}$$
(5) The evaluation model of the comprehensive cognition coordination is constructed as follows:
  (14) $$\begin{array}{c} T=\overset{n}{\underset{\varphi =1}{\sum }}a^{\varphi }Z^{\varphi }. \end{array}$$
(6) Combined with the idea of game theory, the undetermined coefficient *a*^*φ*^ is optimized to minimize the deviation between *Z*^*φ*^ and *Z*^*β*^ as follows:
  (15) $$\begin{array}{c} \min\overset{n}{\underset{\varphi =1}{\sum }}a^{\varphi }Z^{\varphi T}-Z^{\beta },\;\left(\beta =1,\ldots ,n\right). \end{array}$$
According to the differential properties of the matrix, the optimal conditions of formula (15) are obtained as follows:
  (16) $$\begin{array}{c} \overset{n}{\underset{\varphi =1}{\sum }}a^{\varphi }Z^{\beta }Z^{\varphi T}=Z^{\beta }Z^{\beta T}. \end{array}$$
Based on the constraint conditions of combination determining weights on maximizing deviations, the improved optimization model is determined:
  (17) $$\begin{array}{c} \underset{a^{1},a^{2}}{\min}f=\overset{n}{\underset{\beta =1}{\sum }}\left|\left(\overset{n}{\underset{\varphi =1}{\sum }}a^{\varphi }Z^{\beta }Z^{\varphi T}\right)-Z^{\beta }Z^{\beta T}\right|,\\ \text{s.t }a^{\varphi }>0\;\varphi =1,\ldots ,n\;\overset{n}{\underset{\varphi =1}{\sum }}{\left(a^{\varphi }\right)}^{2}=1. \end{array}$$
The Lagrange function is established to solve the model as follows:
  (18) $$\begin{array}{c} L\left(a^{\varphi },\lambda \right)=\overset{n}{\underset{\beta =1}{\sum }}\left|\left(\overset{n}{\underset{\varphi =1}{\sum }}a^{\varphi }Z^{\beta }Z^{\varphi T}\right)-Z^{\beta }Z^{\beta T}\right|+\frac{\lambda }{2}\left(\overset{n}{\underset{\varphi =1}{\sum }}{\left(a^{\varphi }\right)}^{2}-1\right). \end{array}$$
Thus, when the cognition of the two design subjects is closely matched, the solution of the undetermined coefficient is as follows:
  (19) $$\begin{array}{c} a^{\varphi }=\frac{\sum_{\beta =1}^{n}Z^{\beta }Z^{\varphi T}}{\sqrt{\sum_{\varphi =1}^{n}{\left(\sum_{\beta =1}^{n}Z^{\beta }Z^{\varphi T}\right)}^{2}}}. \end{array}$$
The undetermined coefficient is normalized to obtain the optimized undetermined coefficient:
  (20) $$\begin{array}{c} a_{*}^{\varphi }=\frac{a^{\varphi }}{\sum_{\varphi =1}^{n}a^{\varphi }}. \end{array}$$
(7) We substitute the optimized undetermined coefficient into formula (14) and obtain the cognitive matching model of the design subjects as follows:
  (21) $$\begin{array}{c} D=\sqrt{CT}. \end{array}$$
When the cognitive matching degree *D* ≥ 0.75, the cognition of the two design subjects reaches the matching state.

### 3.2. Determination of the Kansei Image Prototype

To make the product evolution operation convenient, based on the results of the Kansei image survey of users in 3.1.1, we take the average value of the survey results as the judgement standard; that is, when $r_{ib}\geq \bar{r_{ib}}$, we select the sample to construct the Kansei image prototype cluster, *U* = [*u*_1_, *u*_*2*_,…, *u*_*h*_], where *u*_*h*_ is a Kansei image prototype. The evaluation value of the Kansei image cognition can reflect the representative degree of product samples to Kansei images; that is, the higher the evaluation value is, the higher the representative degree is, and the higher the user's Kansei image recognition is. Therefore, the Kansei image prototype with the highest cognitive evaluation value is selected as the representative Kansei image prototype.

### 3.3. Product Kansei Image Evaluation System

Based on the sample set, we parameterize the sample contour, train the ANN with the Kansei image survey results, and finally establish the product Kansei image evaluation system with the cognitive matching model of design subjects. The specific process is as follows:

  Step 1: The contours of all samples in the sample set established in 3.1.1 are extracted and parameterized to obtain the key point coordinates.
  Step 2: The coordinates of the key points of the sample are taken as the input, and the Kansei image cognitive evaluation values of users and designers are taken as the output. The ANN is trained to obtain an ANN based on users' cognition and an ANN based on designers' cognition.
  Step 3: The two trained ANNs obtained in Step 2 are combined with the cognitive matching model of the design subjects constructed in 3.1.3 to obtain the product Kansei image evaluation system, which is used as the fitness function of the product evolution system.

### 3.4. Product Kansei Image Form Evolution System

The product Kansei image form evolution system established in this paper is a man-machine interface interaction system based on GA and MATLAB programming. According to the genetic law, we perform genetic operations on the parameterized parent samples and evaluate the new form through a fitness function to obtain a product form with high cognitive matching. The specific process is as follows:

  Step 1: A sample is randomly selected from the sample set to be the product prototype. We input this sample and the representative Kansei image prototype determined in 3.2 into the system as the parents for chromosome coding.
  Step 2: The crossover probability and mutation probability determined in 3.1.2 are the input, and a genetic operation is carried out to obtain the new product form.
  Step 3: The product form generated in step 2 is estimated by the fitness function. If the new form meets the requirements, the product form is output. If the new form does not meet the requirements, all forms are sorted according to the degree of cognitive matching, from which the product form with the highest degree of cognitive matching is selected to replace the product prototype in Step 1, and Steps 1∼3 are repeated until a product form meeting the requirements of the fitness function is obtained to ensure that the product form is always subject to Kansei image traction.
  Step 4: After conducting the Kansei image cognition survey of the design subjects on the output product form, we compare and analyse the results with the results of the product Kansei image evaluation system to verify the feasibility of the method proposed in this paper.

## 4. Case Validation

To prove the feasibility of the research method proposed in this paper, we choose a beverage bottle as an example for verification.

### 4.1. Kansei Image Cognition Experiment

#### 4.1.1. Determination of the Representative Samples

**Figure 3:**
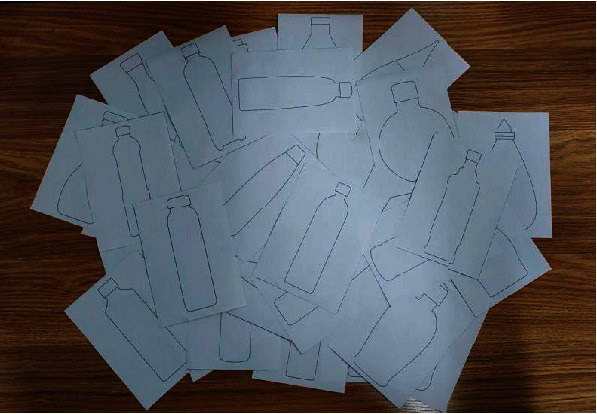
Sample cards.

A total of 130 pictures of beverage bottles are collected from websites, journals, and other channels. After preliminary screening, we obtain 77 sample pictures. To prevent the influence of colour, pattern, and other factors on the Kansei image cognition of the designers and users, we extract and draw the contour lines of all beverage bottles and convert them into 10 × 15 cm cards, as shown in Figure 3.

We invite 5 experts to screen the beverage bottle samples based on morphological differences and obtain 65 representative samples, some of which are shown in Table 1 (see Table S1 in the Supplementary Material for all representative samples).

#### 4.1.2. Determination of the Representative Kansei Words

According to journals, consumer evaluation, and other channels, we collect 46 Kansei image words about the forms of beverage bottles and select 15 Kansei words with the KJ method to construct the Kansei image set. We conduct a semantic similarity survey with 20 experts and import the average value of the survey results into SPSS software for cluster analysis. The clustering results of the Kansei words are shown in Table 2. We select the Kansei image words nearest to the centre as the representative Kansei images, including simplism, comfortable, streamlined, exquisite, and novel.

#### 4.1.3. Survey of the Design Subjects' Kansei Image Cognition and Result Statistics

We create an SD questionnaire with 65 representative samples and 5 representative Kansei words and investigate 54 users and 46 designers. There are 47 and 44 valid questionnaires from each group, respectively. The survey data are analysed, and some results are shown in Tables 3 and 4 (see Tables S3 and S4 in the Supplementary Material for the survey results of the Kansei image cognition of all users and designers).

As the research process of each Kansei image is the same, this paper takes “Exquisite” as an example for verifying the method. Some of the cognitive evaluations of users and designers based on this Kansei image are shown in Table 5 (see Table S5 in the Supplementary Material for all evaluation values of “Exquisite”).

Formula (4) is used to normalize the cognitive evaluation of the two design subjects, and the decision matrix *V* is obtained. Some of the results are shown in Table 6 (see Table S6 in the Supplementary Material for all normalization results).

After averaging the data in Table 6, we can obtain the Kansei image cognition probability matrix of users and designers, as shown in Table 7, to determine the mutation probability and crossover probability.

### 4.2. Cognitive Matching Model Based on the Design Subjects

To build the cognitive matching model based on the design subjects, we use formulas (6)∼(9) to calculate the entropy, weight, and weighted cognitive evaluation of the two design subjects, as shown in Table 8.

We introduce TOPSIS and obtain the relative closeness between the user and the target Kansei image and between the designer and the target Kansei image according to formulas (10)∼(12), as shown in Table 9.

The relative closeness of the two design subjects in Table 9 is introduced into formula (13), and the coordination index *C* = 0.25 of the Kansei image cognition of the two design subjects is obtained.

According to formula (21), there is a strong correlation between the evaluation of the cognitive matching model *D* based on the design subjects and the comprehensive cognitive evaluation model *T*. To make the evaluation results of the cognitive matching model more scientific and reasonable, only if the cognitive evaluation of the two cognitive subjects is closest to the target Kansei image evaluation can the constructed evaluation model of the comprehensive cognition coordination express the Kansei image cognitive evaluation of any cognitive subject. Therefore, based on formulas (15)∼(20), we can obtain the optimized undetermined coefficient *a*_*∗*_^*φ*^, as shown in Table 10.

We substitute the values in Table 10 into formula (14) and coordinate index *C* and formula (14) into formula (21) to obtain the cognitive matching model *D* based on the design subjects.

### 4.3. Determination of the Representative Kansei Image Prototype

According to Table 7 in 4.1.3, the average value of the Kansei image cognitive evaluation of all samples is 0.378. We compare the Kansei image cognitive evaluation of users in Table 6 with the average value of the Kansei image cognitive evaluation and construct a Kansei image prototype cluster for all product forms whose cognitive evaluation is higher than the average value of Kansei image cognitive evaluation to obtain Kansei image prototype cluster *B*. The form and number are shown in Table 11.

**Figure 4:**
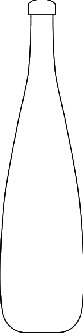
The representative Kansei image prototype.

Among them, the users' Kansei image cognition evaluation value of sample 13 is the highest, so we choose sample 13 as the representative Kansei image prototype, as shown in Figure 4.

### 4.4. Product Kansei Image Form Evaluation System

**Figure 5:**
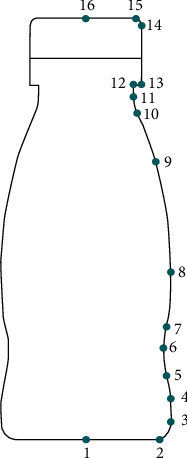
Key points of the beverage bottle contour.

The contours of all samples in Table 1 are parameterized. Because the contours of a beverage bottle are symmetrical, we study half of the bottle. We use 16 key control points to quantify the sample contours, as shown in Figure 5. All key points are represented by coordinates, and some data are shown in Table 12 (see Table S12 in the Supplementary Material for the key point coordinates of all samples).

**Figure 6:**
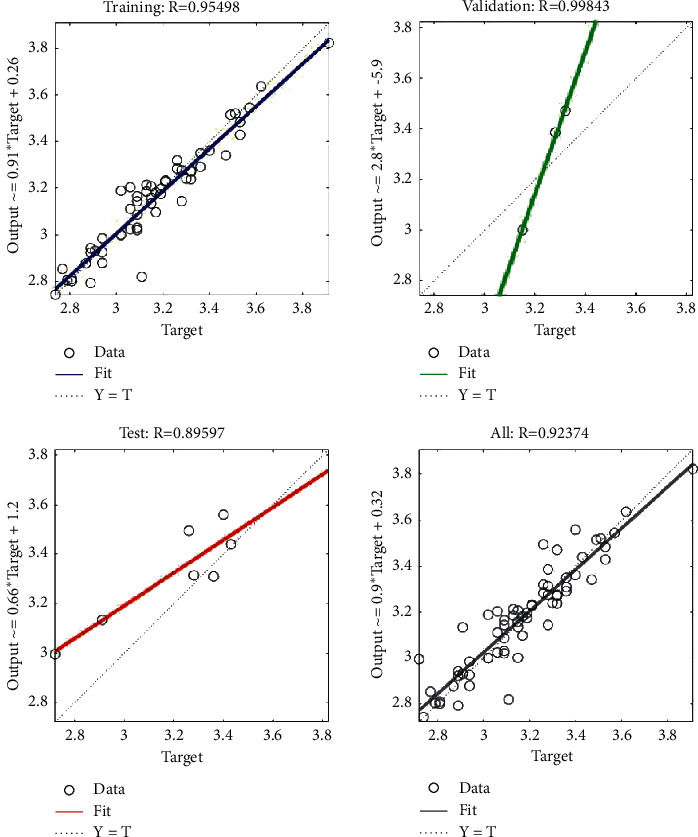
Training results of the ANN based on user cognition.

**Figure 7:**
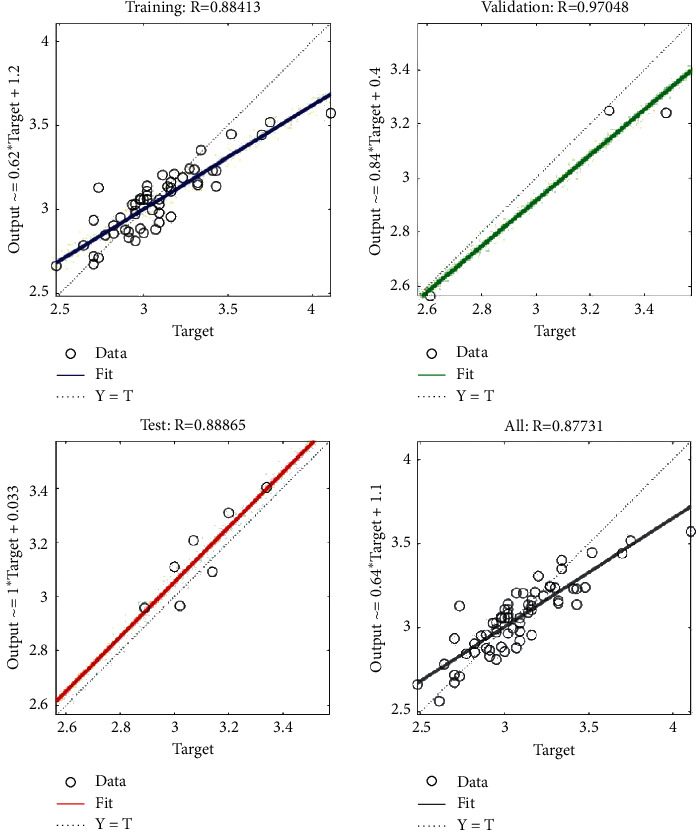
Training results of the ANN based on designer cognition.

We take the data in Table 12 as input and the data in Table 5 as output to train the ANN. The training results are shown in Figures 6 and 7. Figures 6 and 7 show that the overall accuracies of the two ANNs are 92.374% and 87.731%, respectively, and that their errors are less than 0.125. Therefore, we obtain an ANN based on user cognition and an ANN based on designer cognition. The two neural networks are combined with the cognitive matching model in Section 4.2, and this is used as the fitness function of the product evolution system.

### 4.5. Product Kansei Image Form Evolution System

**Figure 8:**
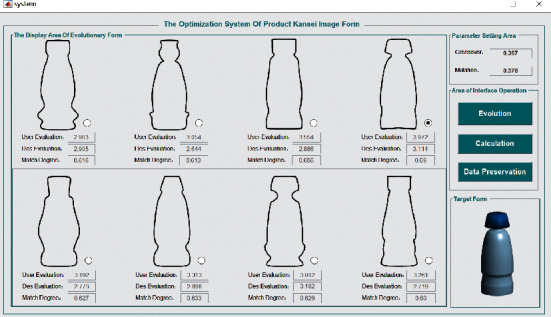
Human-computer interaction interface of the product evolution system.

We select sample 38 from the sample set as the product prototype and form the parent sample with the representative Kansei image prototype. Combining the crossover probability and mutation probability determined in Section 4.1.3, we carry out genetic operation and use the product Kansei image evaluation system obtained in the previous section to judge whether the product form achieves cognitive matching. We use the MATLAB program to present the above operations in the human-computer interaction interface, as shown in Figure 8.

After evolution, the product form with the highest matching degree is selected. We save the coordinates of the key points, replace the product prototype to perform genetic operations again, and repeat until the product form yields a cognitive matching degree *D* ≥ 0.75 between the two design subjects.

**Figure 9:**
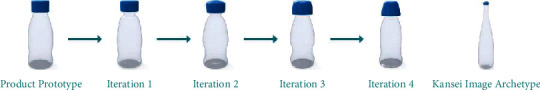
The product evolution process of “Exquisite.”

In this process, the product evolution is led by the Kansei image prototype, and the evolution process is similar to the communication between users and designers. Each iteration of the scheme is similar to the process of cognitive matching between the two design subjects. Because the product form generated by the evolution system is used only as the form reference for the designers in the design and in MATLAB, the linspace function is used for interpolation in the transformation of the two-dimensional form into the three-dimensional model, and the resulting contour of the three-dimensional product model is not smooth. Therefore, we derive the key points of the contours of all evolutionary products, use Rhinoceros software for modelling, and arrange all the schemes in the order of evolution to form the evolution diagram of the Kansei image products, as shown in Figure 9.

**Figure 10:**
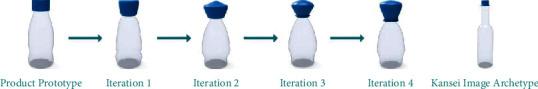
The product evolution process of “Simplism.”

Because in the determination of the Kansei image prototype of the other four Kansei images the construction of the product Kansei image form evaluation system and the product evolution process are the same as the above process, we repeat the process in 4.3∼4.5 to form the product evolution diagram of the other four Kansei images, as shown in Figures 10 ∼ 13.

Although the product prototype and Kansei image prototypes of different Kansei images are constructed under the same conditions, their evolution forms are different due to the different evolution probabilities and evaluation coefficients of the evaluation system. The final product forms of all the Kansei image styles are shown in Table 13.

### 4.6. Verification of the Cognitive Matching Model

**Figure 14:**
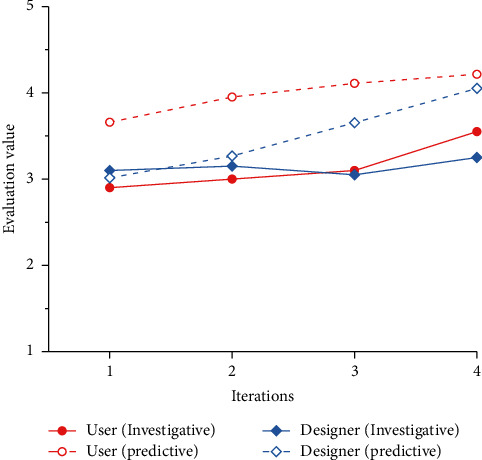
Comparison of the cognitive evaluation under “Exquisite.”

To verify the feasibility of the cognitive matching model proposed in this paper, we combine all the product forms generated by the evolution system with the corresponding Kansei images and invite 20 users and 20 designers to evaluate the Kansei image cognition. The results are shown in Table 14. Combined with the results in Table 14, line charts of the predicted values and survey values of design subjects under different Kansei images are drawn, as shown in Figures 14 ∼18.

### 4.7. Result Analysis

Figures 14∼18 show that, except for “Comfortable,” the predicted results of the other four Kansei images are basically consistent with the trend of the survey results, and the evaluation results basically increase gradually. In addition, with the iteration of the product scheme, the cognitive evaluations of the two design subjects basically cross each other, which is consistent with the matching process of the Kansei image cognition of the two design subjects during the iteration of the product scheme. The cognitive evaluation of the fourth-generation products is basically higher than that of the first three generations, which is consistent with the cognitive evaluation of the two design subjects being higher than that of the iterative process when the cognition achieves a high matching. Therefore, it is proven that our cognitive matching model is feasible and can provide a reference for designers' design activities.

**Figure 17:**
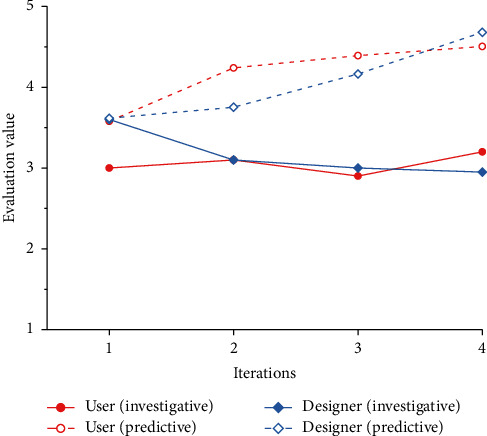
Comparison of the cognitive evaluation under “Comfortable.”

According to Figure 17, there is a great difference between the predicted value and the survey value of the design subjects' cognitive evaluation under “Comfortable.” The possible reasons for this are as follows: (1) Compared with the other four Kansei images, “Comfortable” is more abstract and is often expressed as tactile experience. It is difficult for design subjects to express its visual manifestation, and most design subjects may still regard it as a tactile experience. (2) The product form is the result of the synthesis of multiple Kansei images. Because of the differences in the degree of embodiment of different Kansei images, there are primary and secondary distinctions between Kansei images. When we evaluate the product form of a single Kansei image, the Kansei image may be affected by the primary Kansei image, which makes it difficult for the evolutionary form to reflect the morphological characteristics of the Kansei image, thus gradually reducing the cognitive evaluation of the Kansei image. In addition, the survey results in Figures 14∼18 are lower than the predicted results. This may occur because the previous cognitive survey of this study used two-dimensional product contours, while the cognitive survey of the evolutionary product forms used a three-dimensional model. Different product forms have an impact on the cognition of design subjects. Therefore, in subsequent studies, we will focus on the above issues in depth to improve the accuracy of the cognitive matching model.

In terms of innovation, existing studies, such as those in [19, 20, 28, 29], mainly analyse the cognitive differences from a qualitative level and explore the final product form of cognitive matching of different groups in product design, and they give less consideration to Kansei image traction. Compared with the existing studies, the advantages of cognitive matching model of the design subjects constructed in this paper are as follows:

1. Based on the iteration of a single scheme, the cognitive change trend of users and designers in the process of scheme iteration is discussed quantitatively. This helps designers grasp the cognitive differences between the two cognitive subjects more accurately to improve the acceptance of products.
2. To address the problem that the Kansei image style of the offspring product deviates from the style of the target Kansei image, this paper introduces the concept of Kansei image traction and uses Kansei image prototypes to guide the evolution of the offspring product form to help ensure that the product scheme always has the characteristics of the target Kansei image.
3. The method proposed in this paper can effectively shorten the evaluation cycle of a large number of schemes generated by intelligent design, and it can help designers quickly and accurately select alternative schemes that fit the Kansei image cognition of the two design subjects, thus shortening the product development cycle to a certain extent.

## 5. Discussion

In product design, the Kansei image cognition of users and designers is a factor that cannot be ignored. However, due to their different, complex backgrounds in terms of factors such as education and society, there are differences between users' and designers' cognitions; the designed products reflect the designer's cognition to a greater extent, resulting in low recognition of products by users. The cognitive matching model of design subjects proposed in this paper simulates the process of the cognitive matching of the two design subjects in a quantitative way. By improving the cognitive matching degree of design subjects in evolutionary products, we can reduce the cognitive differences in the process of product design. To some extent, the matching model helps designers understand the cognitive trends of the two design subjects and comprehensively consider their own cognition and users' needs so that a strong matching of the Kansei image cognition of design subjects and the efficient development of products can be achieved. This provides guidance and new research ideas for intelligent product design that balances the cognition of multiple design subjects.

Human cognition of things is a complex process that is affected by many factors such as regional culture, educational background, and the surrounding environment. In this paper, a questionnaire survey is used to obtain the cognition of design subjects, and this process has strong subjectivity. In future research, we will apply more objective methods such as EEG experiments to obtain the Kansei image cognition of design subjects.

The Kansei image prototype is the concrete form of the Kansei image, and morphological fusion needs to consider the cognitive commonality and the topological structure between the two characteristic lines. To facilitate the fusion between forms, the beverage bottle with the highest Kansei image evaluation is regarded as the Kansei image prototype. Future research will focus on the fusion algorithm of different types of forms, thus simplifying the process of morphological fusion.

An accurate product model can more accurately reflect the Kansei image cognition of design subjects. In this paper, the linspace function can roughly reflect the three-dimensional shape of the product, but the contour fluency of the model is not too high. Subsequent research will further explore the interpolation function of the model construction in the three-dimensional modelling software in order to redesign the product Kansei image form evolution system to improve the accuracy of the three-dimensional model.

In the process of product development, all participants, such as users, designers, engineers, design decision-makers, and marketers, are the design subjects of the product. To facilitate this research, the design subjects are only the users and designers in the paper, and other participants such as engineers are not considered. Next, we will introduce other design participants to explore the impact of different participants' interventions on product form.

The evaluation system of this study is based on an ANN, which needs a large number of samples to improve the prediction accuracy. In this study, the sample size used in neural network training is relatively small, which may reduce the accuracy of the evaluation system. Future research will focus on exploring the intelligent evaluation algorithm with a small sample size, using a small sample size in training to improve the recognition performance of the evaluation model.

Finally, colour, material, and surface technology (CMF) are popular research topics at this stage, and the integration of various elements can reflect the cognition of design subjects. However, to improve the accuracy of the cognitive matching model, this paper explores only the cognitive matching process of the product form. Combining CMF to further explore cognitive matching from the overall perspective will be the focus of future research.

## 6. Conclusions

This paper presents a method of cognitive matching of design subjects in product form evolutionary design. First, we explore the inherent trends of the Kansei image cognition of the two design subjects by using the relevant methods of Kansei engineering, and we construct a cognitive matching model of the design subjects. Second, according to the obtained product form that represents the Kansei image, the Kansei image prototype cluster is constructed, and the representative Kansei image prototype is selected as external traction to lead the evolution of the product form. Then, we combine an ANN with the cognitive matching model of the design subjects to construct the product Kansei image evaluation system; this is used as the evaluation mechanism of the evolution system. Finally, a product Kansei image form evolution system is established based on a GA and verified by comparative experiments. The results show that the model can effectively realize the Kansei image cognition of the two design subjects.

The proposed method largely simulates the process of the interaction between designers and users in product design, helps designers to more accurately understand the cognitive trends of the two design subjects, and provides a quantitative method for the cognitive matching of the design subjects, which has guiding significance for the continuation of the evolutionary product Kansei image style in the process of product form evolution. Although we take beverage bottles as an example to study, this model is applicable to the form evolution of other products. Simulating the cognitive matching process of the two design subjects in the iterative process of the product scheme provides a research basis for intelligent product design that balances the cognition of multiple design subjects.

## Figures and Tables

**Figure 11:**
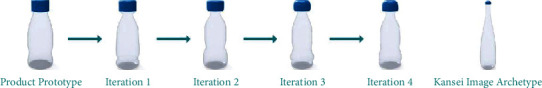
The product evolution process of “Streamlined.”

**Figure 12:**
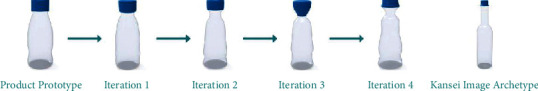
The product evolution process of “Comfortable.”

**Figure 13:**
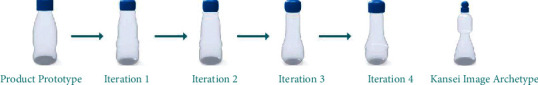
The product evolution process of “Novel.”

**Figure 15:**
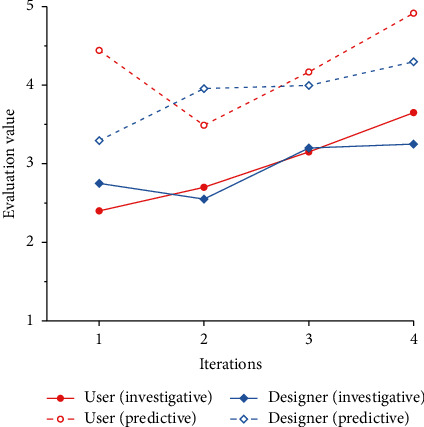
Comparison of the cognitive evaluation under “Simplism.”

**Figure 16:**
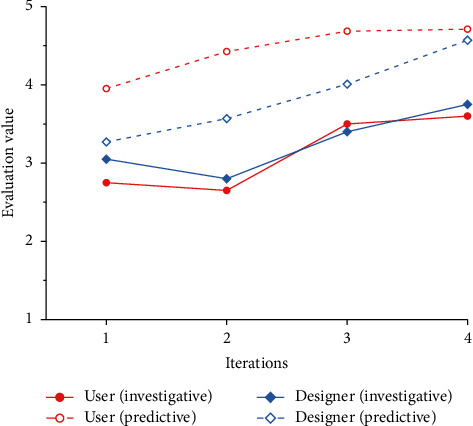
Comparison of the cognitive evaluation under “Streamlined.”

**Figure 18:**
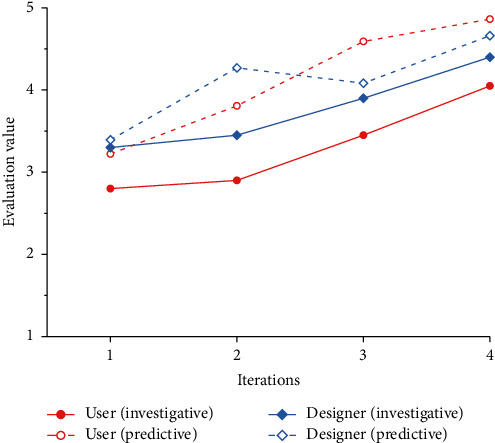
Comparison of the cognitive evaluation under “Novel.”

**Table 1:** Some representative samples.

1	2	3	4	5	6	7
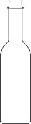	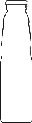	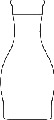	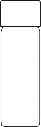	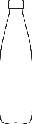	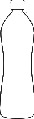	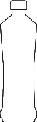
8	9	10	11	12	13	14
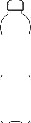	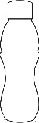	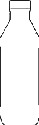	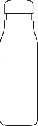	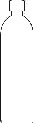	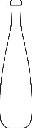	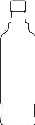
…						
59	60	61	62	63	64	65
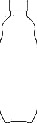	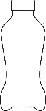	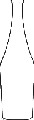	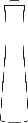	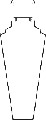	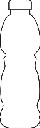	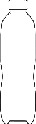

**Table 2:** Clustering results of the Kansei images.

Number	Kansei image	Category	Distance
1	Simplism	1	0.33000
2	Lightweight	1	0.33000
3	Streamlined	3	0.30507
4	Textural	4	0.37350
5	Beautiful	3	0.51592
6	Comfortable	2	0.33439
7	Interesting	5	0.33864
8	Rounded	2	0.45008
9	Sporty	3	0.49180
10	Coordinated	2	0.41757
11	Individual	5	0.29168
12	Friendly	2	0.35619
13	Novel	5	0.24518
14	Exquisite	4	0.37350
15	Fashionable	3	0.44127

**Table 3:** Survey results of the Kansei image cognition of some users.

Sample	Simplism	Comfortable	Streamlined	Exquisite	Novel
1	4.68	4.09	3.21	3.26	1.91
2	3.13	2.57	3.21	2.81	2.83
3	4.02	3.15	3.7	3.36	3.26
4	4.19	3.32	2.72	3.32	2.64
5	4.64	3.85	3.81	3.51	2.4
6	4.13	3.83	3.94	3.49	2.19
7	3.23	2.85	2.62	3.13	2.91
8	3.26	3.04	2.96	3.21	3.06
9	2.19	2.47	2.87	2.87	3.96
10	3.51	3.3	2.79	3.17	2.64
…					
61	4.04	3.55	3.72	3.62	3.19
62	3.98	2.96	3.32	3.26	4.09
63	2.13	2.23	2.47	2.89	4.02
64	2.49	2.4	2.89	2.94	3.57
65	3.7	3.32	3.32	3.32	3

**Table 4:** Survey results of the Kansei image cognition of some designers.

Sample	Simplism	Comfortable	Streamlined	Exquisite	Novel
1	4.32	3.57	2.82	3.02	1.61
2	2.75	2.98	2.91	2.95	3.36
3	3.66	3.34	4.34	3.52	3.61
4	4.61	3.36	2.2	3.14	2.75
5	4.48	3.73	3.91	3.3	2.23
6	3.91	3.75	4.2	3.32	2
7	3.18	2.84	1.89	3.02	3.14
8	2.8	2.91	3.11	3	2.77
9	1.8	2.27	2.98	3.09	4.05
10	3.3	3.09	2.41	2.82	2.86
…					
61	3.8	3.39	3.23	3.27	3.16
62	4.14	3.14	3.07	3.34	4.02
63	1.91	2.16	1.75	2.7	4.2
64	1.7	2.3	2.89	2.95	3.73
65	3.36	3.18	3.36	2.89	2.75

**Table 5:** Some evaluation values of “Exquisite.”

Sample	User	Designer
1	3.26	3.02
2	2.81	2.95
3	3.36	3.52
4	3.32	3.14
5	3.51	3.3
6	3.49	3.32
7	3.13	3.02
8	3.21	3
9	2.87	3.09
10	3.17	2.82
…		
61	3.62	3.27
62	3.26	3.34
63	2.89	2.7
64	2.94	2.95
65	3.32	2.89

**Table 6:** Some of the normalization results.

Sample	User	Designer
1	0.454	0.331
2	0.076	0.288
3	0.538	0.638
4	0.504	0.405
5	0.664	0.503
6	0.647	0.515
7	0.345	0.331
8	0.412	0.319
9	0.126	0.374
10	0.378	0.209
…		
61	0.765	0.485
62	0.454	0.528
63	0.143	0.135
64	0.185	0.288
65	0.504	0.252

**Table 7:** The probability of the Kansei image cognition of the design subjects.

Design subject	Probability of the Kansei image cognition
User	0.378
Designer	0.367

**Table 8:** The entropy, weight, and weighted cognitive evaluation of the two design subjects.

	User	Designer
Entropy	0.963	0.973
Weigh	0.583	0.417
Weighted cognitive evaluation	1.848	1.284

**Table 9:** The relative closeness.

	User	Designer
*d* ^+^	14.88	15.65
*d* ^−^	17.60	16.91
*t* ^*φ*^	0.54	0.52

**Table 10:** The undetermined coefficient of each design subject after evolution.

	User	Designer
*a*_*∗*_^*φ*^	0.59	0.41

**Table 11:** Kansei image prototype cluster.

1	3	4	5	6	8	10
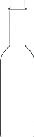	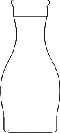	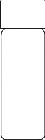	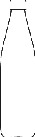	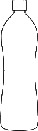	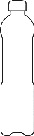	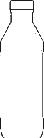
11	12	13	15	17	18	19
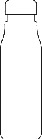	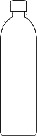	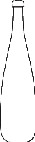	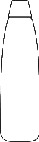	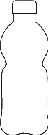	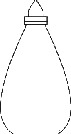	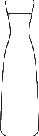
22	26	27	38	39	40	42
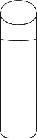	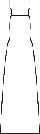	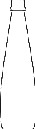	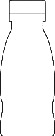	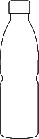	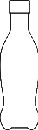	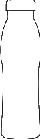
44	48	51	54	55	61	62
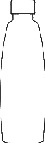	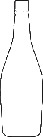	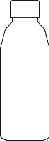	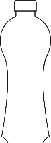	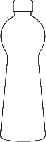	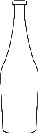	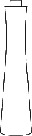
65						
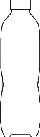						

**Table 12:** Key point coordinates of some samples.

Point and coordinate		Sample 1	Sample 2	Sample 3	Sample 4	Sample 5	Sample 6	Sample 7	Sample 8	Sample 9
1	*x*	0	0	0	0	0	0	0	0	0
	*y*	0	0	0	0	0	0	0	0	0
2	*x*	23.89	26.37	37.15	31	13.47	24.17	34.57	3.81	34.34
	*y*	0.1	0.1	0.1	0.1	-0.55	2.07	3.88	0.1	16.52
3	*x*	32.98	33.9	51.28	41.94	19.82	36.69	35.63	13.4	40.54
	*y*	7.35	11.9	6.09	9.84	-1.56	27.12	11.55	-3.88	42.15
4	*x*	32.98	33.9	51.29	41.94	33.1	33.6	38.54	18.81	34.34
	*y*	51.15	128.86	31.41	52.92	10.01	71.92	12.17	-5.92	75.2
5	*x*	32.98	29.94	54.22	41.94	34.16	30.32	39.16	31.33	31.04
	*y*	105	146.3	49.08	96.31	21.03	102.71	22.49	3.47	102.95
6	*x*	32.98	29.94	57.88	41.94	34.16	29.99	34.35	31.33	31.04
	*y*	148.76	162.31	76.35	147.64	83.29	125.28	37.53	85.73	115.18
7	*x*	20.22	30.6	55.21	41.94	34.16	32.26	33.2	30.39	41.86
	*y*	172.44	174.51	107.56	180.98	129.1	155.17	82.46	97.19	145.05
8	*X*	14.63	26.76	36.17	41.94	31.52	35.1	33.78	28.8	40.45
	*y*	181.33	215.48	151.92	206.11	164.14	179.08	135.73	106.01	150.93
9	*x*	14.63	24.29	28.98	35.32	25.57	31.85	38.01	28.8	40.57
	*y*	218.36	231.83	173.9	218.45	190.18	203.07	212.87	180.09	162.51
10	*x*	14.63	20.12	27.14	43.44	18.93	19.04	16.58	31.46	37.06
	*y*	249.53	245.51	191.56	218.55	217.71	222.65	231.16	194.38	193.36
11	*x*	17.36	20.52	28.98	43.44	15.29	10.76	14.2	13.63	25.16
	*y*	249.53	249.28	211.71	237.07	232.34	231.47	236.45	231.95	220.31
12	*x*	17.36	20.52	32.63	43.44	13.17	10.76	14.2	13.63	25.16
	*y*	250.62	255.32	227.29	254.71	247.06	236.66	243.58	239.54	224.44
13	*x*	13.78	22.6	32.63	43.44	15.29	14.11	17.55	16.32	26.34
	*y*	252.59	256.22	239.85	269.22	247.16	236.76	243.68	239.54	224.54
14	*x*	13.78	22.6	35.79	37.66	15.29	14.11	17.55	16.32	26.34
	*y*	267.29	262.06	250.12	274.73	263.15	253.16	259.29	255.41	257
15	*x*	13.78	29.23	28.99	21.72	10.12	10.76	13.41	10.47	21.85
	*y*	269.06	265.63	259.57	274.63	266.35	256.24	263.34	259.72	262
16	*x*	0	0	0	0	0	0	0	0	0
	*y*	269.16	265.73	259.67	274.73	266.45	256.34	263.44	260.7	264.76

**Table 13:** The evolution forms of the final products from different images.

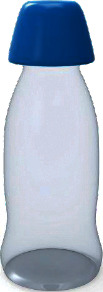	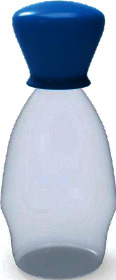	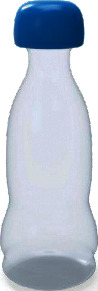	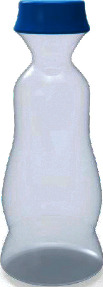	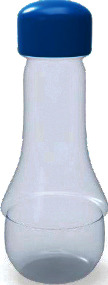
Exquisite	Simplism	Streamlined	Comfortable	Novel

**Table 14:** Comparison of the survey evaluation and predicted evaluation results.

Kansei image	Design subject	1	2	3	4
Exquisite	User (survey)	2.9	3	3.1	3.55
	User (prediction)	3.659	3.951	4.109	4.215
	Designer (survey)	3.1	3.15	3.05	3.25
	Designer (prediction)	3.015	3.267	3.653	4.051
Simplism	User (survey)	2.4	2.7	3.15	3.65
	User (prediction)	4.441	3.489	4.167	4.915
	Designer (survey)	2.75	2.55	3.2	3.25
	Designer (prediction)	3.295	3.956	3.996	4.297
Streamlined	User (survey)	2.75	2.65	3.5	3.6
	User (prediction)	3.951	4.426	4.686	4.712
	Designer (survey)	3.05	2.8	3.4	3.75
	Designer (prediction)	3.27	3.568	4.009	4.57
Comfortable	User (survey)	3	3.1	2.9	3.2
	User (prediction)	3.578	4.239	4.391	4.504
	Designer (survey)	3.6	3.1	3	2.95
	Designer (prediction)	3.616	3.752	4.164	4.68
Novel	User (survey)	2.8	3.222	3.3	3.394
	User (prediction)	2.9	3.807	3.45	4.269
	Designer (survey)	3.45	4.591	3.9	4.083
	Designer (prediction)	4.05	4.863	4.4	4.661

## Data Availability

The data used to support the findings of this study are included within the supplementary information files.

## Supplementary Materials

Supplementary Materials Table S1: all representative samples. Table S3: survey results of the Kansei image cognition of all users. Table S4: survey results of the Kansei image cognition of all designers. Table S5: all evaluation values of “Exquisite.” Table S6: all normalization results. Table S12: key point coordinates of all samples.
