# Supplementary material for: Cognitive Matching of Design Subjects in Product Form Evolutionary Design
Source: Comput Intell Neurosci. 2021 Sep 30;2021:8456736. doi: 10.1155/2021/8456736 (PMC8500774; doi:10.1155/2021/8456736)
Supplement: Supplementary Materials — Table S1: all representative samples. Table S3: survey results of the Kansei image cognition of all users. Table S4: survey results of the Kansei image cognition of all designers. Table S5: all evaluation values of “Exquisite.” Table S6: all normalization results. Table S12: key point coordinates of all samples. [file 8456736.f1.docx]

## Supplementary Materials

Table S1 Supplementary Material for all representative samples

| 1 | 2 | 3 | 4 | 5 | 6 | 7 |
| --- | --- | --- | --- | --- | --- | --- |
| 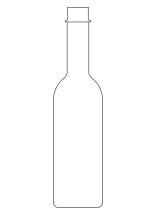 | 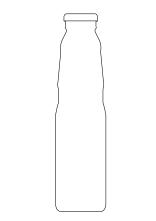 | 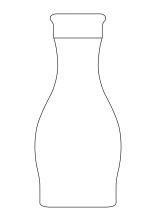 | 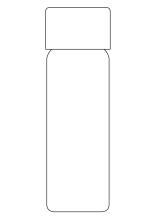 | 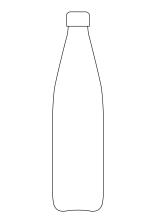 | 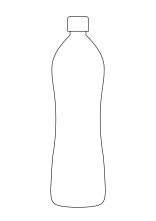 | 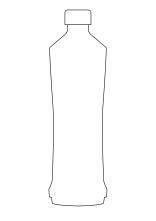 |
| 8 | 9 | 10 | 11 | 12 | 13 | 14 |
| 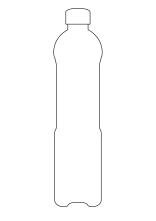 | 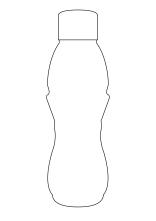 | 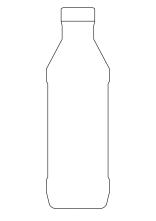 | 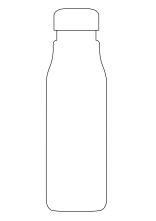 | 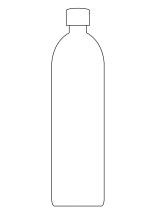 | 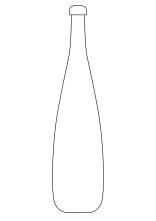 | 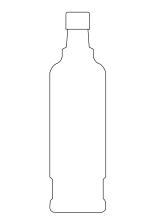 |
| 15 | 16 | 17 | 18 | 19 | 20 | 21 |
| 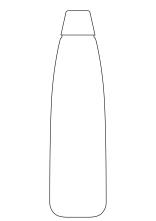 | 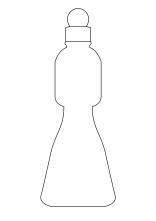 | 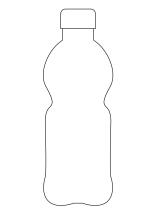 | 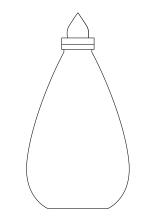 | 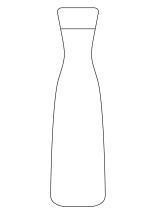 | 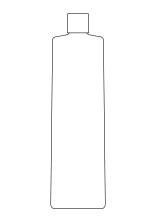 | 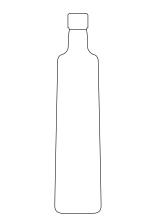 |
| 22 | 23 | 24 | 25 | 26 | 27 | 28 |
| 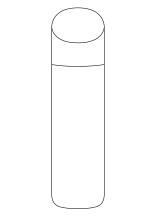 | 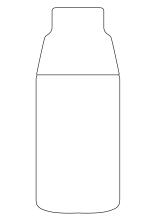 | 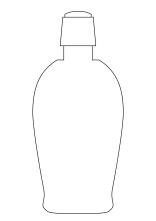 | 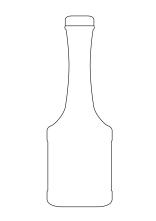 | 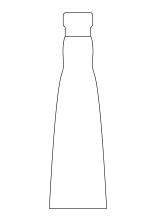 | 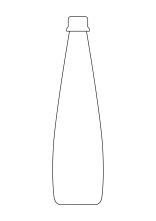 | 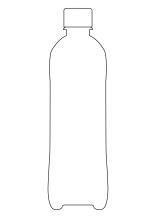 |
| 29 | 30 | 31 | 32 | 33 | 34 | 35 |
| 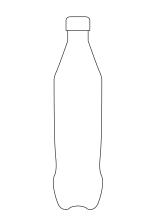 | 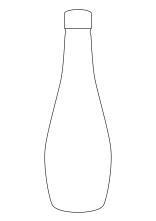 | 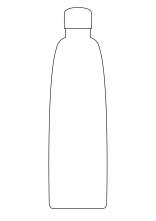 | 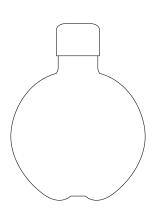 | 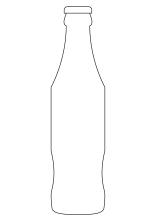 | 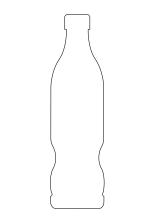 | 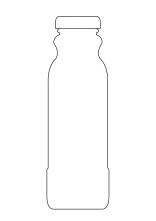 |
| 36 | 37 | 38 | 39 | 40 | 41 | 42 |
| 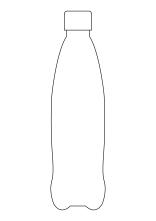 | 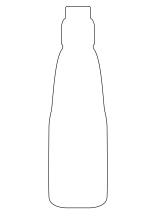 | 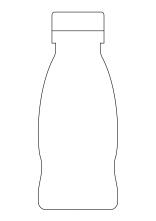 | 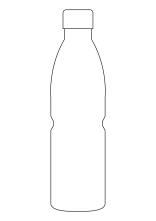 | 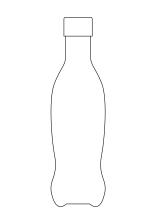 | 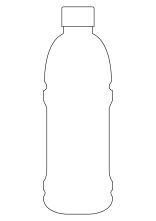 | 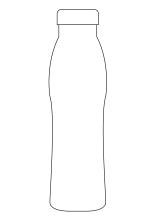 |
| 43 | 44 | 45 | 46 | 47 | 48 | 49 |
| 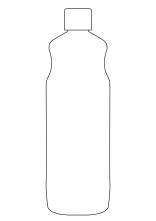 | 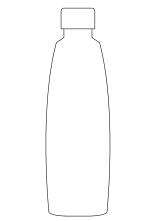 | 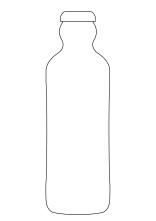 | 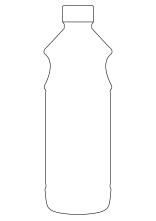 | 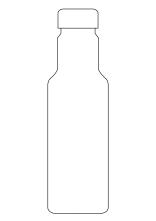 | 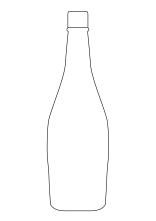 | 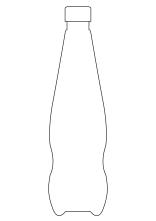 |
| 50 | 51 | 52 | 53 | 54 | 55 | 56 |
| 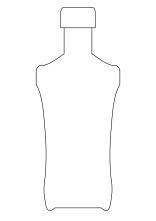 | 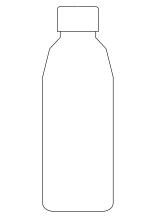 | 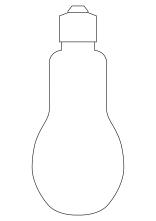 | 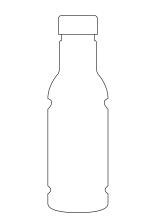 | 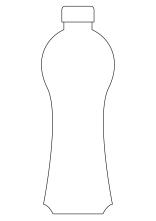 | 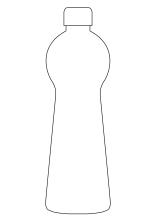 | 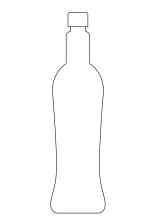 |
| 57 | 58 | 59 | 60 | 61 | 62 | 63 |
| 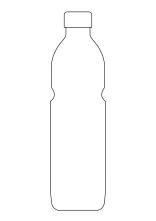 | 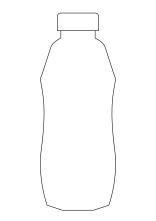 | 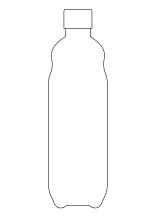 | 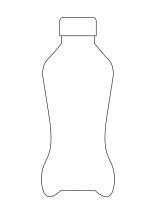 | 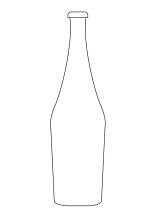 | 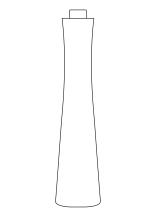 | 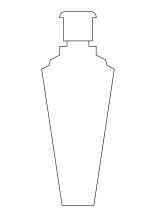 |
| 64 | 65 |  |  |  |  |  |
| 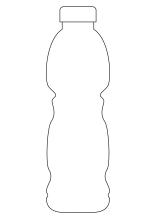 | 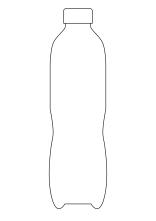 |  |  |  |  |  |

Table S3 Supplementary Material for survey results of the Kansei image cognition of all users

| Sample | Simplism | Comfortable | Streamlined | Exquisite | Novel |
| --- | --- | --- | --- | --- | --- |
| 1 | 4.68 | 4.09 | 3.21 | 3.26 | 1.91 |
| 2 | 3.13 | 2.57 | 3.21 | 2.81 | 2.83 |
| 3 | 4.02 | 3.15 | 3.7 | 3.36 | 3.26 |
| 4 | 4.19 | 3.32 | 2.72 | 3.32 | 2.64 |
| 5 | 4.64 | 3.85 | 3.81 | 3.51 | 2.4 |
| 6 | 4.13 | 3.83 | 3.94 | 3.49 | 2.19 |
| 7 | 3.23 | 2.85 | 2.62 | 3.13 | 2.91 |
| 8 | 3.26 | 3.04 | 2.96 | 3.21 | 3.06 |
| 9 | 2.19 | 2.47 | 2.87 | 2.87 | 3.96 |
| 10 | 3.51 | 3.3 | 2.79 | 3.17 | 2.64 |
| 11 | 3.87 | 3.83 | 3.19 | 3.53 | 2.64 |
| 12 | 4.36 | 3.85 | 3.55 | 3.4 | 2.26 |
| 13 | 4.43 | 3.77 | 4.38 | 3.91 | 3.38 |
| 14 | 1.98 | 2.51 | 2.38 | 2.79 | 3.89 |
| 15 | 4.02 | 3.45 | 3.62 | 3.28 | 3.45 |
| 16 | 1.49 | 1.91 | 2.66 | 2.77 | 4.4 |
| 17 | 3.15 | 3.23 | 3.26 | 3.32 | 3.26 |
| 18 | 3.17 | 2.85 | 3.45 | 3.28 | 3.91 |
| 19 | 3.91 | 3.57 | 3.77 | 3.57 | 3.53 |
| 20 | 4.17 | 3.21 | 2.64 | 3.09 | 2.6 |
| 21 | 4 | 3.34 | 2.85 | 3.13 | 2.89 |
| 22 | 4.04 | 3.45 | 2.98 | 3.4 | 3.19 |
| 23 | 3.21 | 2.85 | 2.79 | 2.81 | 3.28 |
| 24 | 3.06 | 2.85 | 3.32 | 3.02 | 3.15 |
| 25 | 2.81 | 2.53 | 3.13 | 2.91 | 3.55 |
| 26 | 3.89 | 3.36 | 3.17 | 3.36 | 3.06 |
| 27 | 4.23 | 3.83 | 3.87 | 3.36 | 2.79 |
| 28 | 3.72 | 3.26 | 3.3 | 2.89 | 2.77 |
| 29 | 3.68 | 3.4 | 3.21 | 3.06 | 2.45 |
| 30 | 4.15 | 3.85 | 3.87 | 3.47 | 2.98 |
| 31 | 3.77 | 3.15 | 3.11 | 3.06 | 2.89 |
| 32 | 3.4 | 2.68 | 3.43 | 3.11 | 3.79 |
| 33 | 3.32 | 3.02 | 2.94 | 3.09 | 3.09 |
| 34 | 3 | 2.96 | 3.04 | 3.09 | 3.34 |
| 35 | 2.98 | 3.04 | 3 | 3.15 | 3.32 |
| 36 | 3.74 | 3.62 | 3.68 | 3.43 | 2.91 |
| 37 | 3.17 | 2.96 | 2.98 | 2.94 | 3.28 |
| 38 | 3.28 | 2.98 | 3.11 | 3.26 | 3.3 |
| 39 | 3.57 | 3.32 | 3.26 | 3.17 | 3.04 |
| 40 | 3.38 | 3.32 | 3.36 | 3.3 | 3.13 |
| 41 | 2.83 | 2.81 | 2.89 | 3.06 | 3.06 |
| 42 | 3.89 | 3.4 | 3.64 | 3.19 | 2.98 |
| 43 | 3.32 | 2.96 | 3.17 | 3.15 | 3.09 |
| 44 | 4.02 | 3.47 | 3.6 | 3.32 | 3 |
| 45 | 3.47 | 3.02 | 3.32 | 3.02 | 3.19 |
| 46 | 2.79 | 2.49 | 2.64 | 2.94 | 3.55 |
| 47 | 3.55 | 3.3 | 2.98 | 3.15 | 3.04 |
| 48 | 3.96 | 3.68 | 3.74 | 3.53 | 3.17 |
| 49 | 3.51 | 3.11 | 3.47 | 3.02 | 3.32 |
| 50 | 2.4 | 2.19 | 2.62 | 2.74 | 3.77 |
| 51 | 3.6 | 3.13 | 3.11 | 3.28 | 2.94 |
| 52 | 2.85 | 2.68 | 3.3 | 3.09 | 3.94 |
| 53 | 2.72 | 2.64 | 2.74 | 2.91 | 3.47 |
| 54 | 3.26 | 3.09 | 3.06 | 3.19 | 3.4 |
| 55 | 3.7 | 3.17 | 3.38 | 3.28 | 3.13 |
| 56 | 3.34 | 2.85 | 3.13 | 3.09 | 3.17 |
| 57 | 3.51 | 2.91 | 3.13 | 3.15 | 2.87 |
| 58 | 3.06 | 2.85 | 2.94 | 2.72 | 3.09 |
| 59 | 3.4 | 3.19 | 3.21 | 3.21 | 3.04 |
| 60 | 3.04 | 2.68 | 3.11 | 2.89 | 3.53 |
| 61 | 4.04 | 3.55 | 3.72 | 3.62 | 3.19 |
| 62 | 3.98 | 2.96 | 3.32 | 3.26 | 4.09 |
| 63 | 2.13 | 2.23 | 2.47 | 2.89 | 4.02 |
| 64 | 2.49 | 2.4 | 2.89 | 2.94 | 3.57 |
| 65 | 3.7 | 3.32 | 3.32 | 3.32 | 3 |

Table S4 Supplementary Material for survey results of the Kansei image cognition of all designers

| Sample | Simplism | Comfortable | Streamlined | Exquisite | Novel |
| --- | --- | --- | --- | --- | --- |
| 1 | 4.32 | 3.57 | 2.82 | 3.02 | 1.61 |
| 2 | 2.75 | 2.98 | 2.91 | 2.95 | 3.36 |
| 3 | 3.66 | 3.34 | 4.34 | 3.52 | 3.61 |
| 4 | 4.61 | 3.36 | 2.2 | 3.14 | 2.75 |
| 5 | 4.48 | 3.73 | 3.91 | 3.3 | 2.23 |
| 6 | 3.91 | 3.75 | 4.2 | 3.32 | 2 |
| 7 | 3.18 | 2.84 | 1.89 | 3.02 | 3.14 |
| 8 | 2.8 | 2.91 | 3.11 | 3 | 2.77 |
| 9 | 1.8 | 2.27 | 2.98 | 3.09 | 4.05 |
| 10 | 3.3 | 3.09 | 2.41 | 2.82 | 2.86 |
| 11 | 3.68 | 3.61 | 3.18 | 3.43 | 2.98 |
| 12 | 4.48 | 3.5 | 3.43 | 3.05 | 2.41 |
| 13 | 4.59 | 4 | 4.48 | 4.11 | 3.77 |
| 14 | 2.09 | 2.25 | 2.2 | 2.98 | 3.86 |
| 15 | 4.2 | 3.66 | 3.89 | 3.41 | 3.52 |
| 16 | 1.64 | 1.95 | 2.77 | 3.07 | 4.5 |
| 17 | 2.8 | 3.14 | 3.52 | 3.07 | 3.27 |
| 18 | 3.32 | 2.7 | 4 | 3.34 | 4.25 |
| 19 | 3.95 | 3.68 | 3.95 | 3.75 | 3.86 |
| 20 | 4.27 | 3.09 | 2.09 | 3.09 | 3.14 |
| 21 | 3.91 | 3.11 | 2.57 | 3.23 | 3.27 |
| 22 | 4 | 2.86 | 2.48 | 2.98 | 3.32 |
| 23 | 3.64 | 2.75 | 2.77 | 3.02 | 3.52 |
| 24 | 2.8 | 2.52 | 3.59 | 3 | 3.45 |
| 25 | 2.93 | 2.48 | 3.16 | 3.16 | 3.64 |
| 26 | 3.89 | 3.64 | 3.09 | 3.16 | 3.23 |
| 27 | 4.5 | 4.18 | 4.11 | 3.7 | 2.77 |
| 28 | 3.52 | 3.34 | 3.09 | 2.91 | 1.91 |
| 29 | 3.25 | 3.48 | 3.18 | 3.11 | 2 |
| 30 | 4.18 | 3.81 | 4 | 3.48 | 3.36 |
| 31 | 3.68 | 3.07 | 3.25 | 2.86 | 2.8 |
| 32 | 2.91 | 2.39 | 3.52 | 3 | 3.73 |
| 33 | 2.84 | 2.89 | 2.41 | 2.73 | 2.77 |
| 34 | 2.59 | 2.8 | 3.11 | 3.09 | 3.25 |
| 35 | 2.75 | 2.98 | 2.93 | 2.89 | 3.3 |
| 36 | 3.8 | 3.41 | 3.7 | 3.2 | 2.59 |
| 37 | 3 | 2.95 | 2.95 | 3.18 | 3.41 |
| 38 | 3.25 | 2.77 | 3.27 | 2.7 | 2.95 |
| 39 | 3.57 | 3.3 | 3.07 | 3.02 | 2.91 |
| 40 | 3.23 | 3.11 | 3.48 | 3.02 | 2.7 |
| 41 | 2.16 | 2.52 | 2.68 | 2.91 | 2.8 |
| 42 | 3.84 | 3.14 | 3.2 | 3.09 | 3.07 |
| 43 | 3.11 | 2.59 | 2.91 | 2.73 | 3.14 |
| 44 | 4.14 | 3.66 | 3.61 | 3.32 | 3.07 |
| 45 | 3.07 | 2.93 | 3.09 | 3.02 | 3.45 |
| 46 | 2.16 | 2.09 | 2.34 | 2.48 | 3.59 |
| 47 | 3.43 | 3.09 | 2.57 | 3.16 | 2.86 |
| 48 | 3.89 | 3.73 | 4.11 | 3.43 | 3.18 |
| 49 | 3.09 | 3.18 | 3.5 | 3.27 | 3.41 |
| 50 | 1.98 | 2.09 | 2.18 | 2.64 | 3.95 |
| 51 | 3.55 | 2.95 | 2.5 | 2.7 | 2.77 |
| 52 | 2.45 | 2.68 | 3.3 | 3.14 | 3.95 |
| 53 | 2.2 | 2.57 | 2.34 | 2.77 | 3.52 |
| 54 | 2.98 | 2.84 | 3.14 | 2.82 | 3.7 |
| 55 | 3.32 | 3.3 | 3.45 | 3.16 | 3.18 |
| 56 | 2.61 | 2.77 | 3.16 | 2.95 | 3.48 |
| 57 | 3.43 | 3.25 | 2.84 | 2.98 | 2.75 |
| 58 | 3 | 2.7 | 2.64 | 2.61 | 3.39 |
| 59 | 3.34 | 3.09 | 3.07 | 2.93 | 2.89 |
| 60 | 2.73 | 2.5 | 3.2 | 2.95 | 3.5 |
| 61 | 3.8 | 3.39 | 3.23 | 3.27 | 3.16 |
| 62 | 4.14 | 3.14 | 3.07 | 3.34 | 4.02 |
| 63 | 1.91 | 2.16 | 1.75 | 2.7 | 4.2 |
| 64 | 1.7 | 2.3 | 2.89 | 2.95 | 3.73 |
| 65 | 3.36 | 3.18 | 3.36 | 2.89 | 2.75 |

Table S5 Supplementary Material for all evaluation values of 'Exquisite'

| Sample | User | Designer |
| --- | --- | --- |
| 1 | 3.26 | 3.02 |
| 2 | 2.81 | 2.95 |
| 3 | 3.36 | 3.52 |
| 4 | 3.32 | 3.14 |
| 5 | 3.51 | 3.3 |
| 6 | 3.49 | 3.32 |
| 7 | 3.13 | 3.02 |
| 8 | 3.21 | 3 |
| 9 | 2.87 | 3.09 |
| 10 | 3.17 | 2.82 |
| 11 | 3.53 | 3.43 |
| 12 | 3.4 | 3.05 |
| 13 | 3.91 | 4.11 |
| 14 | 2.79 | 2.98 |
| 15 | 3.28 | 3.41 |
| 16 | 2.77 | 3.07 |
| 17 | 3.32 | 3.07 |
| 18 | 3.28 | 3.34 |
| 19 | 3.57 | 3.75 |
| 20 | 3.09 | 3.09 |
| 21 | 3.13 | 3.23 |
| 22 | 3.4 | 2.98 |
| 23 | 2.81 | 3.02 |
| 24 | 3.02 | 3 |
| 25 | 2.91 | 3.16 |
| 26 | 3.36 | 3.16 |
| 27 | 3.36 | 3.7 |
| 28 | 2.89 | 2.91 |
| 29 | 3.06 | 3.11 |
| 30 | 3.47 | 3.48 |
| 31 | 3.06 | 2.86 |
| 32 | 3.11 | 3 |
| 33 | 3.09 | 2.73 |
| 34 | 3.09 | 3.09 |
| 35 | 3.15 | 2.89 |
| 36 | 3.43 | 3.2 |
| 37 | 2.94 | 3.18 |
| 38 | 3.26 | 2.7 |
| 39 | 3.17 | 3.02 |
| 40 | 3.3 | 3.02 |
| 41 | 3.06 | 2.91 |
| 42 | 3.19 | 3.09 |
| 43 | 3.15 | 2.73 |
| 44 | 3.32 | 3.32 |
| 45 | 3.02 | 3.02 |
| 46 | 2.94 | 2.48 |
| 47 | 3.15 | 3.16 |
| 48 | 3.53 | 3.43 |
| 49 | 3.02 | 3.27 |
| 50 | 2.74 | 2.64 |
| 51 | 3.28 | 2.7 |
| 52 | 3.09 | 3.14 |
| 53 | 2.91 | 2.77 |
| 54 | 3.19 | 2.82 |
| 55 | 3.28 | 3.16 |
| 56 | 3.09 | 2.95 |
| 57 | 3.15 | 2.98 |
| 58 | 2.72 | 2.61 |
| 59 | 3.21 | 2.93 |
| 60 | 2.89 | 2.95 |
| 61 | 3.62 | 3.27 |
| 62 | 3.26 | 3.34 |
| 63 | 2.89 | 2.7 |
| 64 | 2.94 | 2.95 |
| 65 | 3.32 | 2.89 |

Table S6 Supplementary Material for all normalization results

| Sample | User | Designer |
| --- | --- | --- |
| 1 | 0.454 | 0.331 |
| 2 | 0.076 | 0.288 |
| 3 | 0.538 | 0.638 |
| 4 | 0.504 | 0.405 |
| 5 | 0.664 | 0.503 |
| 6 | 0.647 | 0.515 |
| 7 | 0.345 | 0.331 |
| 8 | 0.412 | 0.319 |
| 9 | 0.126 | 0.374 |
| 10 | 0.378 | 0.209 |
| 11 | 0.681 | 0.583 |
| 12 | 0.571 | 0.350 |
| 13 | 1 | 1 |
| 14 | 0.059 | 0.307 |
| 15 | 0.471 | 0.571 |
| 16 | 0.042 | 0.362 |
| 17 | 0.504 | 0.362 |
| 18 | 0.471 | 0.528 |
| 19 | 0.714 | 0.779 |
| 20 | 0.311 | 0.374 |
| 21 | 0.345 | 0.460 |
| 22 | 0.571 | 0.307 |
| 23 | 0.076 | 0.331 |
| 24 | 0.252 | 0.319 |
| 25 | 0.160 | 0.417 |
| 26 | 0.538 | 0.417 |
| 27 | 0.538 | 0.748 |
| 28 | 0.143 | 0.264 |
| 29 | 0.286 | 0.387 |
| 30 | 0.630 | 0.613 |
| 31 | 0.286 | 0.233 |
| 32 | 0.328 | 0.319 |
| 33 | 0.311 | 0.153 |
| 34 | 0.311 | 0.374 |
| 35 | 0.361 | 0.252 |
| 36 | 0.597 | 0.442 |
| 37 | 0.185 | 0.429 |
| 38 | 0.454 | 0.135 |
| 39 | 0.378 | 0.331 |
| 40 | 0.487 | 0.331 |
| 41 | 0.286 | 0.264 |
| 42 | 0.395 | 0.374 |
| 43 | 0.361 | 0.153 |
| 44 | 0.504 | 0.515 |
| 45 | 0.252 | 0.331 |
| 46 | 0.185 | 0.001 |
| 47 | 0.361 | 0.417 |
| 48 | 0.681 | 0.583 |
| 49 | 0.252 | 0.485 |
| 50 | 0.017 | 0.098 |
| 51 | 0.471 | 0.135 |
| 52 | 0.311 | 0.405 |
| 53 | 0.160 | 0.178 |
| 54 | 0.395 | 0.209 |
| 55 | 0.471 | 0.417 |
| 56 | 0.311 | 0.288 |
| 57 | 0.361 | 0.307 |
| 58 | 0.001 | 0.080 |
| 59 | 0.412 | 0.276 |
| 60 | 0.143 | 0.288 |
| 61 | 0.765 | 0.485 |
| 62 | 0.454 | 0.528 |
| 63 | 0.143 | 0.135 |
| 64 | 0.185 | 0.288 |
| 65 | 0.504 | 0.252 |

Table S12 Supplementary Material for key point coordinates of all samples

| Point and Coordinate | | Sample 1 | Sample 2 | Sample 3 | Sample 4 | Sample 5 | Sample 6 | Sample 7 | Sample 8 | Sample 9 |
| --- | --- | --- | --- | --- | --- | --- | --- | --- | --- | --- |
| 1 | x | 0 | 0 | 0 | 0 | 0 | 0 | 0 | 0 | 0 |
|  | y | 0 | 0 | 0 | 0 | 0 | 0 | 0 | 0 | 0 |
| 2 | x | 23.89 | 26.37 | 37.15 | 31 | 13.47 | 24.17 | 34.57 | 3.81 | 34.34 |
|  | y | 0.1 | 0.1 | 0.1 | 0.1 | -0.55 | 2.07 | 3.88 | 0.1 | 16.52 |
| 3 | x | 32.98 | 33.9 | 51.28 | 41.94 | 19.82 | 36.69 | 35.63 | 13.4 | 40.54 |
|  | y | 7.35 | 11.9 | 6.09 | 9.84 | -1.56 | 27.12 | 11.55 | -3.88 | 42.15 |
| 4 | x | 32.98 | 33.9 | 51.29 | 41.94 | 33.1 | 33.6 | 38.54 | 18.81 | 34.34 |
|  | y | 51.15 | 128.86 | 31.41 | 52.92 | 10.01 | 71.92 | 12.17 | -5.92 | 75.2 |
| 5 | x | 32.98 | 29.94 | 54.22 | 41.94 | 34.16 | 30.32 | 39.16 | 31.33 | 31.04 |
|  | y | 105 | 146.3 | 49.08 | 96.31 | 21.03 | 102.71 | 22.49 | 3.47 | 102.95 |
| 6 | x | 32.98 | 29.94 | 57.88 | 41.94 | 34.16 | 29.99 | 34.35 | 31.33 | 31.04 |
|  | y | 148.76 | 162.31 | 76.35 | 147.64 | 83.29 | 125.28 | 37.53 | 85.73 | 115.18 |
| 7 | x | 20.22 | 30.6 | 55.21 | 41.94 | 34.16 | 32.26 | 33.2 | 30.39 | 41.86 |
|  | y | 172.44 | 174.51 | 107.56 | 180.98 | 129.1 | 155.17 | 82.46 | 97.19 | 145.05 |
| 8 | x | 14.63 | 26.76 | 36.17 | 41.94 | 31.52 | 35.1 | 33.78 | 28.8 | 40.45 |
|  | y | 181.33 | 215.48 | 151.92 | 206.11 | 164.14 | 179.08 | 135.73 | 106.01 | 150.93 |
| 9 | x | 14.63 | 24.29 | 28.98 | 35.32 | 25.57 | 31.85 | 38.01 | 28.8 | 40.57 |
|  | y | 218.36 | 231.83 | 173.9 | 218.45 | 190.18 | 203.07 | 212.87 | 180.09 | 162.51 |
| 10 | x | 14.63 | 20.12 | 27.14 | 43.44 | 18.93 | 19.04 | 16.58 | 31.46 | 37.06 |
|  | y | 249.53 | 245.51 | 191.56 | 218.55 | 217.71 | 222.65 | 231.16 | 194.38 | 193.36 |
| 11 | x | 17.36 | 20.52 | 28.98 | 43.44 | 15.29 | 10.76 | 14.2 | 13.63 | 25.16 |
|  | y | 249.53 | 249.28 | 211.71 | 237.07 | 232.34 | 231.47 | 236.45 | 231.95 | 220.31 |
| 12 | x | 17.36 | 20.52 | 32.63 | 43.44 | 13.17 | 10.76 | 14.2 | 13.63 | 25.16 |
|  | y | 250.62 | 255.32 | 227.29 | 254.71 | 247.06 | 236.66 | 243.58 | 239.54 | 224.44 |
| 13 | x | 13.78 | 22.6 | 32.63 | 43.44 | 15.29 | 14.11 | 17.55 | 16.32 | 26.34 |
|  | y | 252.59 | 256.22 | 239.85 | 269.22 | 247.16 | 236.76 | 243.68 | 239.54 | 224.54 |
| 14 | x | 13.78 | 22.6 | 35.79 | 37.66 | 15.29 | 14.11 | 17.55 | 16.32 | 26.34 |
|  | y | 267.29 | 262.06 | 250.12 | 274.73 | 263.15 | 253.16 | 259.29 | 255.41 | 257 |
| 15 | x | 13.78 | 29.23 | 28.99 | 21.72 | 10.12 | 10.76 | 13.41 | 10.47 | 21.85 |
|  | y | 269.06 | 265.63 | 259.57 | 274.63 | 266.35 | 256.24 | 263.34 | 259.72 | 262 |
| 16 | x | 0 | 0 | 0 | 0 | 0 | 0 | 0 | 0 | 0 |
|  | y | 269.16 | 265.73 | 259.67 | 274.73 | 266.45 | 256.34 | 263.44 | 260.7 | 264.76 |

Continued Table S12 Supplementary Material for key point coordinates of all samples

| Point and Coordinate | | Sample 10 | Sample 11 | Sample 12 | Sample 13 | Sample 14 | Sample 15 | Sample 16 | Sample 17 | Sample 18 |
| --- | --- | --- | --- | --- | --- | --- | --- | --- | --- | --- |
| 1 | x | 0 | 0 | 0 | 0 | 0 | 0 | 0 | 0 | 0 |
|  | y | 0 | 0 | 0 | 0 | 0 | 0 | 0 | 0 | 0 |
| 2 | x | 30.56 | 32.95 | 31.04 | 15.05 | 29.46 | 13.87 | 34.92 | 5.88 | 39.96 |
|  | y | 0.1 | 1.82 | 0.1 | 0.1 | 0.1 | 0.1 | 0.1 | 0.1 | 0.1 |
| 3 | x | 38.68 | 40.53 | 35.28 | 32.69 | 37.66 | 29.68 | 49.74 | 23.45 | 64.97 |
|  | y | 6.61 | 13.99 | 10.02 | 18.43 | 7.29 | 3.35 | 15.52 | -3.15 | 28.52 |
| 4 | x | 42.56 | 40.65 | 35.28 | 33.76 | 34.13 | 37.97 | 36.22 | 37.98 | 68.65 |
|  | y | 22.61 | 53.66 | 42.36 | 72.22 | 18.49 | 19.23 | 74.44 | 1.06 | 63.15 |
| 5 | x | 42.56 | 40.63 | 35.28 | 29.52 | 34.13 | 37.76 | 38.69 | 44.21 | 61.29 |
|  | y | 38.6 | 91.92 | 100.04 | 121.27 | 44.51 | 72.26 | 95.6 | 35.16 | 102.3 |
| 6 | x | 40.56 | 40.58 | 35.28 | 24.97 | 37.67 | 36.92 | 19.05 | 44.21 | 48.91 |
|  | y | 42.36 | 137.99 | 135.32 | 148.25 | 47.86 | 121.87 | 134.41 | 109.54 | 141.85 |
| 7 | x | 40.56 | 40.53 | 35.28 | 18.98 | 37.67 | 35.33 | 31.04 | 38.98 | 40.72 |
|  | y | 108.92 | 173.27 | 165.83 | 178.21 | 187.38 | 161.4 | 148.28 | 128.06 | 163.19 |
| 8 | x | 40.56 | 35.86 | 35.28 | 11.88 | 26.94 | 30.29 | 31.04 | 35.01 | 32.58 |
|  | y | 167.83 | 194.62 | 191.35 | 211.93 | 200.83 | 207.26 | 194.38 | 137.65 | 182.21 |
| 9 | x | 42.56 | 31.77 | 26.72 | 9.71 | 21.05 | 23.68 | 15.52 | 43.22 | 18.79 |
|  | y | 171.24 | 210.24 | 218.81 | 235.33 | 211.1 | 239.89 | 213.67 | 156.63 | 210.37 |
| 10 | x | 42.56 | 31.27 | 11.76 | 9.05 | 21.05 | 21.57 | 15.52 | 42.03 | 18.79 |
|  | y | 187.59 | 223.54 | 232.74 | 259.38 | 219.31 | 241.83 | 216.52 | 193.27 | 213.8 |
| 11 | x | 21.3 | 26.33 | 11.76 | 9.05 | 15.29 | 21.57 | 17.8 | 20.99 | 21.85 |
|  | y | 227.57 | 230.42 | 242.76 | 265.54 | 222.37 | 244.73 | 218.8 | 218.9 | 213.9 |
| 12 | x | 21.3 | 26.33 | 15.75 | 11.17 | 13.64 | 22.98 | 16.93 | 20.99 | 21.85 |
|  | y | 245.92 | 243.73 | 242.86 | 265.64 | 246.26 | 244.83 | 221 | 234.5 | 220.32 |
| 13 | x | 22.69 | 30.21 | 15.75 | 11.17 | 15.99 | 19.68 | 16.93 | 23.45 | 13.71 |
|  | y | 251.91 | 243.83 | 252.97 | 266.79 | 246.36 | 259.65 | 240.48 | 234.6 | 220.42 |
| 14 | x | 22.69 | 30.21 | 15.76 | 11.17 | 15.99 | 16.1 | 10.94 | 23.45 | 11.83 |
|  | y | 268.49 | 264.29 | 262.38 | 273.31 | 266.11 | 275.7 | 242.01 | 255.06 | 245.42 |
| 15 | x | 20.22 | 14.34 | 11.76 | 7.84 | 13.17 | 12.92 | 13.76 | 16.93 | 5.71 |
|  | y | 267.92 | 272.66 | 264.8 | 277.06 | 269.19 | 277.36 | 255.59 | 260.78 | 254.96 |
| 16 | x | 0 | 0 | 0 | 0 | 0 | 0 | 0 | 0 | 0 |
|  | y | 270.02 | 272.76 | 265.9 | 278.43 | 269.29 | 277.46 | 266.23 | 260.88 | 263.29 |

Continued Table S12 Supplementary Material for key point coordinates of all samples

| Point and Coordinate | | Sample 19 | Sample 20 | Sample 21 | Sample 22 | Sample 23 | Sample 24 | Sample 25 | Sample 26 | Sample 27 |
| --- | --- | --- | --- | --- | --- | --- | --- | --- | --- | --- |
| 1 | x | 0 | 0 | 0 | 0 | 0 | 0 | 0 | 0 | 0 |
|  | y | 0 | 0 | 0 | 0 | 0 | 0 | 0 | 0 | 0 |
| 2 | x | 12.41 | 32.25 | 14.42 | 10.69 | 12.35 | 18.74 | 23.79 | 19.27 | 24.16 |
|  | y | 0.1 | 0.1 | 0.1 | 0.35 | 0.1 | 0.1 | 0.1 | 0.1 | 0.1 |
| 3 | x | 26.4 | 36.73 | 31.18 | 21.74 | 40.37 | 40.75 | 43.84 | 38.53 | 29.91 |
|  | y | 2.02 | 6.72 | 9.44 | 1.88 | 2.09 | 2.8 | 10.67 | 0 | 3.49 |
| 4 | x | 36.75 | 36.16 | 30.59 | 35.39 | 56.36 | 47.27 | 43.84 | 36.18 | 32.35 |
|  | y | 15.43 | 55.17 | 47.64 | 19.99 | 19.26 | 13.03 | 20.99 | 42.85 | 11.25 |
| 5 | x | 35.86 | 35.8 | 29.49 | 35.39 | 56.36 | 46.57 | 42.68 | 32.32 | 33.98 |
|  | y | 57.52 | 87.39 | 102.6 | 48.68 | 57 | 34.37 | 21.87 | 98.94 | 37.1 |
| 6 | x | 34.96 | 35.36 | 28.31 | 35.39 | 56.36 | 55.61 | 42.68 | 26.62 | 33.42 |
|  | y | 82.19 | 127.38 | 158.41 | 79.73 | 92.44 | 86.77 | 87.14 | 156.14 | 73.09 |
| 7 | x | 33.5 | 34.93 | 27.67 | 35.39 | 56.36 | 61.03 | 43.84 | 21.61 | 27.45 |
|  | y | 110.54 | 166.42 | 188.04 | 109.89 | 138.06 | 138.44 | 89.61 | 182.04 | 129.34 |
| 8 | x | 30.81 | 34.46 | 27.27 | 35.39 | 56.36 | 50.93 | 43.84 | 19.47 | 21.94 |
|  | y | 143.64 | 208.74 | 206.14 | 139.35 | 180.01 | 183.42 | 102.57 | 190.74 | 167.57 |
| 9 | x | 26.28 | 34.26 | 21.98 | 35.39 | 54.6 | 28.22 | 38.42 | 19.27 | 15.48 |
|  | y | 170.77 | 227.09 | 213.2 | 170.57 | 185.89 | 206.43 | 108.83 | 195.84 | 208.64 |
| 10 | x | 19.69 | 29.43 | 13.4 | 35.39 | 55.6 | 19.23 | 19.05 | 20.15 | 11.43 |
|  | y | 194.14 | 230.97 | 221.55 | 193.68 | 186.89 | 206.53 | 134.85 | 214.48 | 232.96 |
| 11 | x | 18.52 | 11.74 | 11.51 | 35.39 | 44.25 | 19.23 | 14.4 | 17.18 | 15.29 |
|  | y | 207.64 | 235.96 | 247.18 | 211.31 | 236.81 | 225.48 | 197.91 | 231.51 | 232.86 |
| 12 | x | 19.71 | 14.38 | 12.34 | 35.39 | 38.69 | 23.99 | 18.27 | 17.18 | 15.29 |
|  | y | 219.23 | 236.06 | 249.2 | 225.07 | 241.5 | 225.58 | 238.13 | 243.48 | 232.96 |
| 13 | x | 23.38 | 14.38 | 13.51 | 35.39 | 34.84 | 20.64 | 19.34 | 19.92 | 13.17 |
|  | y | 238.87 | 249.9 | 249.3 | 243.77 | 250.1 | 263.93 | 243.24 | 246.09 | 238.74 |
| 14 | x | 27.43 | 14.38 | 13.51 | 35.39 | 34.84 | 17.82 | 19.34 | 19.92 | 13.17 |
|  | y | 260.11 | 260.66 | 266.59 | 257.75 | 272.2 | 264.03 | 251.97 | 261.06 | 248.58 |
| 15 | x | 21.08 | 11.21 | 10.34 | 20.79 | 29.48 | 17.82 | 10.23 | 17.7 | 9.26 |
|  | y | 267.07 | 262.15 | 268.6 | 265.84 | 276.01 | 267.74 | 256.46 | 263.04 | 252.58 |
| 16 | x | 0 | 0 | 0 | 0 | 0 | 0 | 0 | 0 | 0 |
|  | y | 267.17 | 262.25 | 268.7 | 269.88 | 276.11 | 271.97 | 256.56 | 263.14 | 252.68 |

Continued Table S12 Supplementary Material for key point coordinates of all samples

| Point and Coordinate | | Sample 28 | Sample 29 | Sample 30 | Sample 31 | Sample 32 | Sample 33 | Sample 34 | Sample 35 | Sample 36 |
| --- | --- | --- | --- | --- | --- | --- | --- | --- | --- | --- |
| 1 | x | 0 | 0 | 0 | 0 | 0 | 0 | 0 | 0 | 0 |
|  | y | 0 | 0 | 0 | 0 | 0 | 0 | 0 | 0 | 0 |
| 2 | x | 11.58 | 18.08 | 17.38 | 33.24 | 10.02 | 10.45 | 28.75 | 33.43 | 10.35 |
|  | y | -3.18 | 1.74 | 0.1 | 0.1 | 0.1 | 0.1 | 0.1 | 0.1 | 0.1 |
| 3 | x | 16.6 | 35.06 | 21.62 | 38.77 | 24.7 | 25.83 | 35.87 | 41.8 | 21.04 |
|  | y | -4.85 | 11.14 | -2.65 | 8.23 | -5.67 | 1.64 | 9.29 | 17.08 | -5.31 |
| 4 | x | 27.63 | 41.04 | 29.73 | 39.21 | 48.12 | 35.03 | 35.87 | 41.8 | 32.34 |
|  | y | 5.06 | 34.87 | -6.17 | 55.97 | 3.65 | 8.88 | 16.58 | 53.18 | 2.29 |
| 5 | x | 33.18 | 44.23 | 37.49 | 38.95 | 69.15 | 35.79 | 34.4 | 40.13 | 36.61 |
|  | y | 32.54 | 60.48 | 0 | 97.37 | 24.38 | 30.4 | 20.99 | 55.3 | 22.53 |
| 6 | x | 30.99 | 43.85 | 38.2 | 37.67 | 84.17 | 32.4 | 32.46 | 40.13 | 34.93 |
|  | y | 42.57 | 88.66 | 67.47 | 141.34 | 49.85 | 53.45 | 54.06 | 170.83 | 34.98 |
| 7 | x | 29.39 | 36.03 | 37.73 | 34.32 | 90.46 | 36.33 | 29.02 | 29.81 | 34.52 |
|  | y | 57.57 | 125.69 | 166.16 | 186.77 | 80.32 | 85.67 | 68.95 | 202.41 | 70.03 |
| 8 | x | 30.16 | 29.58 | 35.37 | 30.19 | 77.2 | 36.22 | 33.04 | 32.1 | 36.04 |
|  | y | 68.56 | 151.18 | 208.26 | 211.2 | 126.64 | 171.16 | 84.08 | 222.34 | 116.29 |
| 9 | x | 32.16 | 22.36 | 15.88 | 27.92 | 55.62 | 18.6 | 35.63 | 24.69 | 35.05 |
|  | y | 169.45 | 181.56 | 227.54 | 219.2 | 151.63 | 223.36 | 94.63 | 234.51 | 150.11 |
| 10 | x | 29.1 | 19.68 | 15.88 | 24.33 | 28.84 | 15.86 | 35.54 | 24.69 | 27.1 |
|  | y | 180.27 | 204.01 | 235.73 | 225.23 | 164.98 | 253.59 | 164.48 | 236.86 | 196.45 |
| 11 | x | 13.7 | 18.56 | 20.03 | 16.78 | 26.72 | 16.12 | 24.34 | 27.95 | 15.64 |
|  | y | 221.9 | 218.92 | 235.83 | 229.98 | 170.86 | 268.76 | 200.73 | 239.34 | 227.37 |
| 12 | x | 13.7 | 17.51 | 20.03 | 16.78 | 26.72 | 16.12 | 14.64 | 24.69 | 15.64 |
|  | y | 235.67 | 237.77 | 236.8 | 241.97 | 188.4 | 270.47 | 240.85 | 242.74 | 236.26 |
| 13 | x | 16.05 | 17.98 | 19.15 | 18.46 | 28.96 | 18.61 | 16.93 | 30.16 | 18.31 |
|  | y | 235.77 | 247.41 | 239.18 | 242.07 | 188.5 | 270.57 | 240.95 | 242.74 | 236.36 |
| 14 | x | 16.05 | 17.98 | 19.15 | 18.46 | 28.96 | 18.61 | 16.93 | 30.16 | 18.31 |
|  | y | 249.53 | 268.11 | 261.58 | 261.73 | 223.9 | 275.2 | 257.53 | 251.71 | 258.94 |
| 15 | x | 9.7 | 12.43 | 14.83 | 12.49 | 14.85 | 13.04 | 12.35 | 24.43 | 15.11 |
|  | y | 254.29 | 271.25 | 264.31 | 268.49 | 231.79 | 279.29 | 260.96 | 259.02 | 261.84 |
| 16 | x | 0 | 0 | 0 | 0 | 0 | 0 | 0 | 0 | 0 |
|  | y | 254.24 | 272.64 | 264.41 | 270.2 | 231.89 | 279.39 | 261.06 | 259.12 | 261.94 |

Continued Table S12 Supplementary Material for key point coordinates of all samples

| Point and Coordinate | | Sample 37 | Sample 38 | Sample 39 | Sample 40 | Sample 41 | Sample 42 | Sample 43 | Sample 44 | Sample 45 |
| --- | --- | --- | --- | --- | --- | --- | --- | --- | --- | --- |
| 1 | x | 0 | 0 | 0 | 0 | 0 | 0 | 0 | 0 | 0 |
|  | y | 0 | 0 | 0 | 0 | 0 | 0 | 0 | 0 | 0 |
| 2 | x | 15.61 | 45.92 | 11.55 | 15.76 | 15.82 | 12.88 | 10.99 | 14.41 | 11.87 |
|  | y | 0.1 | 0.1 | 0.1 | 0.1 | 0.1 | 0.1 | 0.1 | 0.1 | 0.1 |
| 3 | x | 31.85 | 53.5 | 28.22 | 23.6 | 46.74 | 29.76 | 40.51 | 28.9 | 31.45 |
|  | y | 3.8 | 11.02 | 2.31 | -3.57 | 22.9 | 2.85 | 10.17 | 1.15 | 2.18 |
| 4 | x | 39.07 | 53.5 | 36.51 | 34.92 | 46.74 | 37.57 | 42.98 | 39.1 | 42.93 |
|  | y | 14.29 | 25.4 | 10.32 | 11.05 | 32.91 | 11.99 | 42.1 | 5.17 | 20.78 |
| 5 | x | 39.41 | 50.77 | 36.51 | 36.49 | 46.74 | 35.66 | 42.77 | 40.85 | 42.91 |
|  | y | 66.15 | 40.39 | 108.66 | 20.68 | 43.36 | 53.53 | 108.66 | 27.25 | 43.45 |
| 6 | x | 39.07 | 48.89 | 33.07 | 34.92 | 41.98 | 34.22 | 42.98 | 42.57 | 41.88 |
|  | y | 111.3 | 57.56 | 115.01 | 30.57 | 59.56 | 99.2 | 174.04 | 61.84 | 95.13 |
| 7 | x | 36.07 | 50.77 | 33.07 | 30.39 | 41.98 | 34.69 | 42.63 | 43.07 | 42.91 |
|  | y | 129.82 | 71 | 120.91 | 52.39 | 142.82 | 133.06 | 176.42 | 83.02 | 177.33 |
| 8 | x | 30.8 | 53.5 | 26.51 | 31.99 | 46.74 | 37.57 | 41.22 | 41.3 | 42.91 |
|  | y | 178.99 | 105.6 | 127.62 | 67.5 | 153.63 | 154.22 | 183.61 | 152.88 | 192.97 |
| 9 | x | 22.25 | 43.91 | 36.51 | 36.46 | 46.74 | 38.66 | 38.92 | 35.71 | 26.21 |
|  | y | 212.14 | 175.36 | 153.81 | 123.63 | 165.73 | 181.03 | 222.19 | 201.95 | 216.96 |
| 10 | x | 20.39 | 32.1 | 29.41 | 31.99 | 45.04 | 33.17 | 33.63 | 29.1 | 21.89 |
|  | y | 216.77 | 205.32 | 206.34 | 161.45 | 177.98 | 215.01 | 235.24 | 232.95 | 234.74 |
| 11 | x | 22.23 | 29.99 | 18.26 | 16.7 | 19.76 | 25.52 | 14.41 | 18.87 | 20.6 |
|  | y | 220.31 | 215.78 | 230.95 | 199.08 | 242.59 | 234.42 | 246.53 | 240.01 | 258.03 |
| 12 | x | 22.23 | 29.99 | 18.26 | 16.7 | 19.76 | 25.52 | 14.41 | 18.87 | 20.6 |
|  | y | 242.45 | 223.44 | 245.17 | 225.44 | 252.02 | 250.55 | 248.55 | 251.55 | 259.25 |
| 13 | x | 16.93 | 35.04 | 20.17 | 19.05 | 21.7 | 27.87 | 18.02 | 22.75 | 23.33 |
|  | y | 254.71 | 223.54 | 245.27 | 225.54 | 252.12 | 250.65 | 248.65 | 251.65 | 259.35 |
| 14 | x | 16.93 | 35.04 | 20.17 | 19.05 | 21.7 | 27.87 | 18.02 | 22.75 | 23.33 |
|  | y | 267.76 | 260.82 | 266.41 | 248.24 | 276.64 | 263.82 | 274.84 | 275.28 | 265.79 |
| 15 | x | 14.11 | 31.51 | 15.64 | 16.35 | 19.14 | 21.52 | 14.23 | 18.7 | 16.63 |
|  | y | 270.75 | 265.19 | 270.66 | 250.73 | 278.48 | 271.01 | 277.24 | 279.42 | 272.39 |
| 16 | x | 0 | 0 | 0 | 0 | 0 | 0 | 0 | 0 | 0 |
|  | y | 270.85 | 265.29 | 270.76 | 250.83 | 278.58 | 271.11 | 277.34 | 279.52 | 272.49 |

Continued Table S12 Supplementary Material for key point coordinates of all samples

| Point and Coordinate | | Sample 46 | Sample 47 | Sample 48 | Sample 49 | Sample 50 | Sample 51 | Sample 52 | Sample 53 | Sample 54 |
| --- | --- | --- | --- | --- | --- | --- | --- | --- | --- | --- |
| 1 | x | 0 | 0 | 0 | 0 | 0 | 0 | 0 | 0 | 0 |
|  | y | 0 | 0 | 0 | 0 | 0 | 0 | 0 | 0 | 0 |
| 2 | x | 8.26 | 30.52 | 18.46 | 16.99 | 31.31 | 30.61 | 4.12 | 6.53 | 33.62 |
|  | y | 0.1 | 0.1 | 0.1 | 0.1 | 0.1 | 0.1 | 0.1 | 0.1 | 0.1 |
| 3 | x | 34.95 | 40.66 | 29.63 | 25.29 | 37.81 | 47.55 | 39.99 | 40.48 | 38.43 |
|  | y | 5 | 8.11 | 2.11 | -5.34 | -3.66 | 12 | 13.41 | 12.44 | 7.64 |
| 4 | x | 44.48 | 40.66 | 37.16 | 33.93 | 43.13 | 47.55 | 53.39 | 40.48 | 46.44 |
|  | y | 33.69 | 50.27 | 10.7 | 3.88 | 3.18 | 47.27 | 33.28 | 25.31 | 13.05 |
| 5 | x | 43.57 | 40.66 | 38.92 | 37.45 | 45.81 | 47.55 | 61.74 | 40.48 | 38.79 |
|  | y | 40.48 | 118.71 | 44.92 | 49.57 | 24.93 | 80.67 | 62.79 | 35.72 | 61.01 |
| 6 | x | 43.57 | 40.66 | 41.43 | 33.66 | 44.71 | 47.55 | 58.1 | 40.48 | 35.22 |
|  | y | 163.28 | 173.27 | 83.02 | 66.23 | 38.28 | 107.48 | 94.96 | 138.99 | 96.06 |
| 7 | x | 43.95 | 37.92 | 38.75 | 37.45 | 44.28 | 47.55 | 38.69 | 40.48 | 34.17 |
|  | y | 167.51 | 180.75 | 124.3 | 88.9 | 102.08 | 130.76 | 140.64 | 153.19 | 123.83 |
| 8 | x | 44.48 | 27.43 | 31.19 | 33.75 | 56.36 | 47.55 | 38.69 | 28.05 | 41.15 |
|  | y | 174.27 | 195.85 | 147.34 | 147.87 | 166.04 | 158.77 | 202.73 | 184.77 | 168.98 |
| 9 | x | 44.48 | 27.43 | 22 | 25.13 | 56.36 | 47.55 | 22.11 | 25.05 | 49.17 |
|  | y | 215.25 | 216.08 | 174.43 | 195.12 | 185.33 | 180.62 | 221.6 | 193.76 | 199.87 |
| 10 | x | 43.42 | 27.43 | 16.87 | 17.05 | 50.95 | 38.02 | 22.11 | 25.05 | 37.65 |
|  | y | 223.37 | 236.18 | 197.67 | 241.71 | 191.44 | 218.72 | 232.72 | 227.45 | 233.31 |
| 11 | x | 18.61 | 22.96 | 14.88 | 14.79 | 18.73 | 23.77 | 24.94 | 22.14 | 18.92 |
|  | y | 249.94 | 242.06 | 222.01 | 244.48 | 207.2 | 234.01 | 232.74 | 231.25 | 247.65 |
| 12 | x | 18.61 | 22.96 | 12.99 | 14.79 | 18.73 | 23.77 | 24.94 | 22.14 | 18.92 |
|  | y | 255.71 | 249.02 | 241.06 | 255.53 | 241.91 | 241.91 | 267.64 | 239.17 | 257.78 |
| 13 | x | 20.84 | 26.96 | 13.52 | 17.39 | 23.19 | 27.67 | 13.47 | 26.11 | 21.69 |
|  | y | 258.53 | 249.12 | 246.12 | 255.63 | 242.01 | 242.01 | 272.05 | 239.27 | 257.88 |
| 14 | x | 20.84 | 26.96 | 13.52 | 17.39 | 23.19 | 27.67 | 13.47 | 26.11 | 21.69 |
|  | y | 278.75 | 270.17 | 263.29 | 273.05 | 263.41 | 272.11 | 274.17 | 260.61 | 273.99 |
| 15 | x | 18.14 | 19.43 | 11.99 | 12.8 | 19.2 | 21.32 | 8 | 21.52 | 16.45 |
|  | y | 280.53 | 274.77 | 267.66 | 275.38 | 269.19 | 275.77 | 280.95 | 264.66 | 279.06 |
| 16 | x | 0 | 0 | 0 | 0 | 0 | 0 | 0 | 0 | 0 |
|  | y | 280.63 | 274.87 | 267.76 | 275.48 | 269.29 | 275.87 | 281.05 | 264.76 | 279.16 |

Continued Table S12 Supplementary Material for key point coordinates of all samples

| Point and Coordinate | | Sample 55 | Sample 56 | Sample 57 | Sample 58 | Sample 59 | Sample 60 | Sample 61 | Sample 62 | Sample 63 |
| --- | --- | --- | --- | --- | --- | --- | --- | --- | --- | --- |
| 1 | x | 0 | 0 | 0 | 0 | 0 | 0 | 0 | 0 | 0 |
|  | y | 0 | 0 | 0 | 0 | 0 | 0 | 0 | 0 | 0 |
| 2 | x | 12.94 | 28.34 | 26.99 | 20.96 | 15.17 | 23.4 | 13.17 | 10.11 | 21.4 |
|  | y | 0.1 | 0.1 | 0.1 | 0.1 | 0.1 | 0.1 | 0.1 | 0.1 | 0.1 |
| 3 | x | 34.43 | 35.87 | 35.57 | 41.6 | 23.43 | 37.91 | 31.99 | 27.83 | 30.58 |
|  | y | 3.06 | 18.76 | 10.29 | 6.77 | -3.33 | -7.96 | 7.73 | 3.85 | 61.08 |
| 4 | x | 43.36 | 29.49 | 37.19 | 50.32 | 32.63 | 48.21 | 32.65 | 30.22 | 41.36 |
|  | y | 17.84 | 49.31 | 39.25 | 52.21 | 4.41 | 5.29 | 25.1 | 8.16 | 132.77 |
| 5 | x | 38.69 | 29.46 | 37.19 | 49.45 | 38.9 | 45.98 | 33.81 | 27.97 | 48.92 |
|  | y | 68.36 | 88.61 | 146.46 | 81.12 | 20.92 | 33.87 | 49.79 | 36.56 | 183.09 |
| 6 | x | 33.65 | 31.44 | 31.92 | 45.9 | 39.69 | 36.31 | 35.4 | 24.76 | 39.04 |
|  | y | 120.97 | 124.39 | 155 | 120.67 | 36.16 | 70.86 | 82.3 | 80.29 | 188.03 |
| 7 | x | 30.22 | 34.22 | 35.45 | 48.21 | 39.69 | 39.22 | 36.81 | 22.1 | 37.28 |
|  | y | 156.69 | 161.34 | 164.39 | 161.73 | 159.81 | 102.01 | 110.51 | 116.64 | 195.09 |
| 8 | x | 30.84 | 15.88 | 35.45 | 47.51 | 37.75 | 46.8 | 30.46 | 19.4 | 24.46 |
|  | y | 169.1 | 200.85 | 195.44 | 185.36 | 175.68 | 149.99 | 135.55 | 151.22 | 201.97 |
| 9 | x | 35.4 | 15.88 | 27.05 | 36.92 | 35.1 | 46.63 | 22.28 | 18.33 | 24.46 |
|  | y | 178.8 | 215.14 | 219.9 | 217.99 | 195.44 | 165.57 | 160.39 | 177.33 | 213.61 |
| 10 | x | 43.01 | 15.88 | 18.6 | 32.16 | 34.9 | 40.8 | 13.39 | 18.31 | 14.7 |
|  | y | 206.35 | 238.95 | 230.72 | 225.4 | 207.49 | 176.62 | 201.5 | 208.58 | 213.61 |
| 11 | x | 16.11 | 9.88 | 16.2 | 23.08 | 16.76 | 21.93 | 10.09 | 19.92 | 14.7 |
|  | y | 245.83 | 244.77 | 235.74 | 231.49 | 227.01 | 198.5 | 233.97 | 239.08 | 220.84 |
| 12 | x | 16.11 | 9.88 | 16.2 | 23.08 | 16.76 | 21.93 | 10.82 | 21.4 | 19.46 |
|  | y | 253.02 | 250.31 | 245.7 | 241.35 | 237.85 | 208.1 | 252.81 | 253.51 | 220.84 |
| 13 | x | 18.93 | 13.41 | 18.6 | 27.84 | 18.87 | 24.55 | 13.41 | 11.4 | 19.46 |
|  | y | 253.12 | 250.41 | 245.8 | 241.45 | 237.95 | 208.2 | 252.91 | 253.51 | 252.06 |
| 14 | x | 18.93 | 13.41 | 18.6 | 27.84 | 18.87 | 24.55 | 13.41 | 11.4 | 24.46 |
|  | y | 271.23 | 266.99 | 263.17 | 259.98 | 260.88 | 227.07 | 256.68 | 263.51 | 252.05 |
| 15 | x | 13.99 | 10.58 | 14.46 | 23.34 | 16.55 | 20.7 | 8.94 | 10.07 | 16.64 |
|  | y | 277.01 | 269.36 | 266.48 | 264.9 | 263.51 | 232.44 | 260.51 | 264.75 | 260.07 |
| 16 | x | 0 | 0 | 0 | 0 | 0 | 0 | 0 | 0 | 0 |
|  | y | 277.11 | 269.46 | 266.58 | 265 | 263.61 | 232.54 | 260.61 | 264.85 | 260.17 |

Continued Table S12 Supplementary Material for key point coordinates of all samples

| Point and Coordinate | | Sample 64 | Sample 65 |  |  |  |  |  |  |  |
| --- | --- | --- | --- | --- | --- | --- | --- | --- | --- | --- |
| 1 | x | 0 | 0 |  |  |  |  |  |  |  |
|  | y | 0 | 0 |  |  |  |  |  |  |  |
| 2 | x | 20.46 | 17.61 |  |  |  |  |  |  |  |
|  | y | 0.1 | 0.1 |  |  |  |  |  |  |  |
| 3 | x | 42.39 | 25.68 |  |  |  |  |  |  |  |
|  | y | 19.52 | -6.38 |  |  |  |  |  |  |  |
| 4 | x | 41.69 | 34.9 |  |  |  |  |  |  |  |
|  | y | 25.75 | 6.79 |  |  |  |  |  |  |  |
| 5 | x | 37.98 | 38.75 |  |  |  |  |  |  |  |
|  | y | 41.39 | 45.86 |  |  |  |  |  |  |  |
| 6 | x | 39.28 | 36.1 |  |  |  |  |  |  |  |
|  | y | 57.74 | 77.73 |  |  |  |  |  |  |  |
| 7 | x | 35.98 | 33.48 |  |  |  |  |  |  |  |
|  | y | 122.06 | 97.13 |  |  |  |  |  |  |  |
| 8 | x | 34.22 | 37.51 |  |  |  |  |  |  |  |
|  | y | 145.23 | 127.35 |  |  |  |  |  |  |  |
| 9 | x | 30.81 | 37.51 |  |  |  |  |  |  |  |
|  | y | 155.46 | 188.03 |  |  |  |  |  |  |  |
| 10 | x | 39.98 | 32.69 |  |  |  |  |  |  |  |
|  | y | 169.57 | 217.55 |  |  |  |  |  |  |  |
| 11 | x | 21.28 | 17.17 |  |  |  |  |  |  |  |
|  | y | 245.89 | 238.13 |  |  |  |  |  |  |  |
| 12 | x | 21.28 | 17.17 |  |  |  |  |  |  |  |
|  | y | 255.78 | 242.73 |  |  |  |  |  |  |  |
| 13 | x | 24.34 | 19.41 |  |  |  |  |  |  |  |
|  | y | 255.88 | 242.83 |  |  |  |  |  |  |  |
| 14 | x | 24.34 | 19.41 |  |  |  |  |  |  |  |
|  | y | 272.11 | 259.64 |  |  |  |  |  |  |  |
| 15 | x | 19.76 | 15.64 |  |  |  |  |  |  |  |
|  | y | 276.24 | 262.48 |  |  |  |  |  |  |  |
| 16 | x | 0 | 0 |  |  |  |  |  |  |  |
|  | y | 276.34 | 262.58 |  |  |  |  |  |  |  |
